# Activity-Based Protein Profiling for the Identification of Novel Carbohydrate-Active Enzymes Involved in Xylan Degradation in the Hyperthermophilic Euryarchaeon *Thermococcus* sp. Strain 2319x1E

**DOI:** 10.3389/fmicb.2021.734039

**Published:** 2022-01-12

**Authors:** Thomas Klaus, Sabrina Ninck, Andreas Albersmeier, Tobias Busche, Daniel Wibberg, Jianbing Jiang, Alexander G. Elcheninov, Kseniya S. Zayulina, Farnusch Kaschani, Christopher Bräsen, Herman S. Overkleeft, Jörn Kalinowski, Ilya V. Kublanov, Markus Kaiser, Bettina Siebers

**Affiliations:** ^1^Molecular Enzyme Technology and Biochemistry (MEB), Environmental Microbiology and Biotechnology (EMB), Faculty of Chemistry, Centre for Water and Environmental Research (CWE), University of Duisburg-Essen, Essen, Germany; ^2^Department of Chemical Biology, Center of Medical Biotechnology, Faculty of Biology, University of Duisburg-Essen, Essen, Germany; ^3^Center for Biotechnology (CeBiTec), Bielefeld University, Bielefeld, Germany; ^4^Section of Bio-Organic Synthesis, Leiden Institute of Chemistry, Leiden University, Leiden, Netherlands; ^5^Winogradsky Institute of Microbiology, Research Center of Biotechnology, Russian Academy of Sciences, Moscow, Russia

**Keywords:** activity-based protein profiling, archaea, *Thermococcus*, xylan, hemicellulose degradation, glycoside hydrolases

## Abstract

Activity-based protein profiling (ABPP) has so far scarcely been applied in Archaea in general and, especially, in extremophilic organisms. We herein isolated a novel *Thermococcus* strain designated sp. strain 2319x1E derived from the same enrichment culture as the recently reported *Thermococcus* sp. strain 2319x1. Both strains are able to grow with xylan as the sole carbon and energy source, and for *Thermococcus* sp. strain 2319x1E (optimal growth at 85°C, pH 6–7), the induction of xylanolytic activity in the presence of xylan was demonstrated. Since the solely sequence-based identification of xylanolytic enzymes is hardly possible, we established a complementary approach by conducting comparative full proteome analysis in combination with ABPP using α- or β-glycosidase selective probes and subsequent mass spectrometry (MS)-based analysis. This complementary proteomics approach in combination with recombinant protein expression and classical enzyme characterization enabled the identification of a novel bifunctional maltose-forming α-amylase and deacetylase (EGDIFPOO_00674) belonging to the GH57 family and a promiscuous β-glycosidase (EGIDFPOO_00532) with β-xylosidase activity. We thereby further substantiated the general applicability of ABPP in archaea and expanded the ABPP repertoire for the identification of glycoside hydrolases in hyperthermophiles.

## Introduction

Activity-based protein profiling (ABPP) is a powerful technique for the class-specific detection of active enzymes. With the help of an activity-based probe (ABP) that is composed of a reactive inhibitor group, a linker and a reporter group, a target enzyme is covalently modified at the active site and can thereby be detected or identified in downstream analyses (Cravatt et al., 2008). ABPP has been proven as an elaborate tool for several mesophilic enzyme families, allowing to unravel the activity state of enzymes under different conditions without knowledge of their natural substrates or enzyme functions, and thus also helps to deduce their function in certain cellular processes (Cravatt et al., 2008; Willems et al., 2014). This methodology can be applied to various types of biological samples and is even suitable for the *in vivo* study of enzyme activities under native conditions (Speers and Cravatt, 2004).

Recently, its scope of application has been expanded to the identification of serine hydrolases in extremophilic Archaea using phosphonate-derived ABPs (Zweerink et al., 2017). Labeling of α- and β-specific retaining glycoside hydrolases, on the other hand, has been successfully conducted in different Eukaryotes (like mammals, fungi and plants) by employing ABPs, which were designed to react with the active site nucleophiles of retaining glycosidases to form a covalent and irreversible bond (Wu et al., 2019). The two ABPs used in this work, JJB384 and JJB111, are based on two epimeric cyclitol aziridines. JJB384 is composed of 1,6-epi-cyclophellitol aziridine having a biotin moiety attached to the aziridine nitrogen (N_2_). It is a structural isostere of an α-glucopyranoside warhead and thanks to this feature preferably targets retaining α-glucosidases (Jiang et al., 2016). JJB111, the biotinylated, *N*-alkylated aziridine analog of the natural product cyclophellitol, structurally and configurationally resembles β-glucopyranosides and preferentially reacts with β-glucosidases (Kallemeijn et al., 2012). However, neither of the two ABPs are fully in-class selective. JJB384 was found to label, besides α-glucosidases, also several β-glucosidases (Husaini et al., 2018). JJB111 in turn showed activity toward a variety of β-glycosidases, including β-xylosidases such as a bifunctional α-L-arabinofuranosidase/β-D-xylosidase, β-galactosidases, and β-glucuronidases (Chandrasekar et al., 2014; Husaini et al., 2018).

The heteromorphic polysaccharide xylan belongs to the hemicelluloses and, together with other polymers such as cellulose and lignin, is a major constituent of the plant cell wall (Ebringerová and Heinze, 2000). It is therefore one of the most abundant polysaccharides found in nature, and moreover, it is believed to account for about one third of the Earth’s renewable organic carbon (Prade, 1996). In plant cell walls, xylans and other hemicelluloses, such as mannans and galactans, are covalently linked to lignin layers and non-covalently to cellulose fibers (Biely, 1985; Heredia et al., 1995). Xylan structures in plants are highly variable and depend on the phylogenetic position of the species and on their position in primary or secondary cell walls (Harris and Stone, 2008). Many xylans are thereby composed of a linear backbone of 1,4-linked β-D-xylopyranosyl residues. In cell walls, they form heteroxylans with side chains containing other monosaccharides, such as α-L-arabinofuranose and 4-*O*-methyl-α-D-glucuronic acid or α-D-glucuronic acid, as well as oligosaccharides, acetyl groups and, in some taxa, phenolic acid esters, such as ferulate or *p*-coumarate esters (Peña et al., 2016). In hardwood xylan, for instance, some D-xylose molecules are linked to 4-*O*-methylglucuronic acids at the C2 position and are highly acetylated at the C2 and C3 positions. Xylans from softwoods have an even higher content of 4-*O*-methylglucuronic acids, but instead of acetylation, α-L-arabinofuranose units are linked to C3 atoms of the D-xylose backbone (Puls, 1997). For algae and seaweed species, xylans have only been found in small amounts and feature a different structure: β-1,3-linked D-xylose backbones or mixtures of β-1,3 and β-1,4 bonds with diverse side chains (Usov, 2011; Hsieh and Harris, 2019).

**FIGURE 1:**
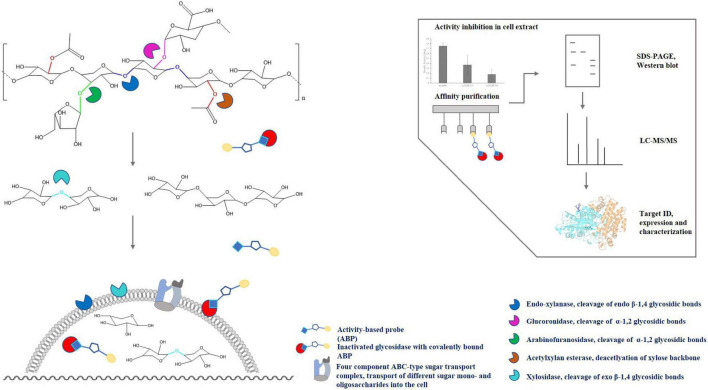
Activity-based protein profiling for the identification of enzymes involved in xylan degradation in *Thermococcus* sp. strain 2319x1E. The schematic structure of hardwood xylan and the enzymes most commonly involved in its degradation as well as the chemical proteomics workflow is depicted. ABPs covalently and irreversibly modify active retaining glycosidases. The thus labeled and inactivated enzymes can be visualized on a gel/membrane or affinity purified via the linked biotin moiety (yellow circle) and subsequently analyzed by LC-MS/MS, followed by protein identification through matching the acquired spectra with a reference database.

Due to the complex and heterogeneous structure of xylan, the complete enzymatic hydrolysis into its monomeric sugars requires different classes of hydrolases such as endo-β-1,4-xylanases (EC 3.2.1.8), β-xylosidases (EC 3.2.1.37), α-glucuronidases (EC 3.2.1.139), α-arabinofuranosidases (EC 3.2.1.55) and furthermore acetylxylan esterases (EC 3.1.1.6) for deacetylation of the xylan backbone and released sugar moieties (Figure 1). Endo-β-1,4-xylanases and β-xylosidases are collectively referred to as xylanases since they are required for hydrolysis of the xylan backbone into D-xylose monomers (Juturu and Wu, 2012). According to the CAZy database (see the Carbohydrate Active Enzymes database^1^; Henrissat et al., 1989; Lombard et al., 2014), all so far reported endoxylanases and β-xylosidases are grouped into 9 and 14 different GH families, respectively. However, most of them originate from fungi or bacteria, while only a few archaeal (hyper)thermophilic xylanases are known so far, that mostly belong to the GH10 and GH11 families (Collins et al., 2005; Thomas et al., 2017). The presence of xylanolytic enzymes has been demonstrated only for a few representatives of the Crenarchaeota and Euryarchaeota, however, examples for a proper characterization of xylan degrading enzymes from those phyla remain scarce (Niehaus et al., 1999; Uhl and Daniel, 1999; Cannio et al., 2004; Collins et al., 2005). In *Thermococcus zilligii*, a xylanolytic enzyme was purified from the culture supernatant and described as the first archaeal hemicellulase discovered, with an activity of 5.07 U mg^–1^ protein using oat spelt xylan as substrate (Uhl and Daniel, 1999). However, *T. zilligii* is not able to grow on xylan. Hence, the recently described Archaeon *Thermococcus* sp. strain 2319x1 (Gavrilov et al., 2016) and a new *Thermococcus* isolate designated sp. strain 2319x1E described in this work, both originating from the same enrichment culture, are the first Euryarchaeota known to be able to utilize xylan as sole carbon and energy source. We thus applied (chemo)proteomics, i.e., ABPP using glycosidase-selective ABPs complemented by a comparative full-proteome analysis, for the identification of xylan degrading enzymes from the new isolate. The respective workflow of ABPP-based chemical proteomics as well as the degradation of the xylan polymer by different classes of enzymes are depicted in Figure 1. This approach extends the repertoire of ABPP in archaea, and hyperthermophiles in particular, by the identification of glycoside hydrolases, highlighting the importance of ABPP for deciphering metabolic processes and identifying novel enzymes.

## Materials and Methods

### Chemicals

Chemicals for cultivation of *Escherichia coli* and *Thermococcus* sp. strain 2319x1E, such as yeast extract, lysogeny broth (LB), beechwood xylan, D-glucose and D-xylose were obtained from Carl Roth (Germany). Avicel^®^ cellulose, carboxymethyl cellulose (CMC), maltodextrin and soluble starch were purchased from Sigma-Aldrich (United States), xylobiose, *ortho*-nitrophenyl-β-D-galactopyranoside (ONPG), *para*-nitrophenyl acetate (PNPA), *para*-nitrophenyl-β-D-xylopyranoside (PNPX) and *para*-nitrophenyl- β-D-glucopyranoside (PNPG) from Megazyme (Ireland). Other chemicals which were used in this work are 4’,6-diamidino-2-phenylindole (DAPI; Thermo Fisher Scientific, United States), urea (GE Healthcare Life Sciences, Germany), ammonium bicarbonate (ABC; Sigma-Aldrich, United States), dithiothreitol (DTT; Sigma-Aldrich, United States), iodoacetamide (IAM; Sigma-Aldrich, United States), formic acid (FA; Fischer Chemical, Germany) and bovine serum albumin (BSA; VWR Chemicals, United States).

### Genome Sequencing, Assembly and Annotation

*Thermococcus* sp. strain 2319x1E was sequenced based on short-read and long-read sequencing methods. In a first step, whole-genome-shotgun PCR-free libraries for Illumina sequencing were constructed from 2 μg of gDNA with the Illumina TruSeq DNA PCR-free Sample Preparation Kit (Illumina, United States) based on the manufacturer’s protocol. The libraries were quality controlled by analysis on an Agilent 2000 Bioanalyzer with Agilent High Sensitivity DNA Kit (Agilent Technologies, United States) for fragment sizes of 550 bp. Sequencing was performed on a Illumina MiSeq (2 × 300 bp/v3 chemistry) in paired-end mode. Adapters and low-quality reads were removed by an in-house software pipeline prior to polishing as recently described (Wibberg et al., 2016).

In a second step, the Oxford Nanopore sequencing library from *Thermococcus* sp. strain 2319x1E gDNA was prepared using the Nanopore Native barcoding genomic DNA kit with native barcodes (SQK-LSK109 with EXP-NBD104) according to the manufacturer’s instructions. The resulting libraries were sequenced on an Oxford Nanopore GridION Mk1 sequencer using an R9.4.1 flow cell, which was prepared according to the manufacturer’s instructions. MinKNOW was used to control the run using the 24 h sequencing run protocol; live base calling was performed using guppy v3.2.6. The assembly was performed as described recently with smaller modifications - Unicycler v0.4.6. was used for the hybrid-assembly and no additional polishing was performed (Wibberg et al., 2020). The finished genome sequence was imported into the annotation platforms Prokka (Seemann, 2014) and GenDB (Meyer et al., 2003) for automatic prediction of genes and functional annotation as described previously (Tomazetto et al., 2018).

### Phylogenetic Analysis

For an extensive phylogenic analysis, all available genomes of cultivated species of *Thermococcus*, *Pyrococcus*, *Palaeococcus*, and *Methanothermobacter thermautotrophicus* Delta H (used as an outgroup) were downloaded from GenBank. In total, 122 archaeal-specific conserved proteins were identified from 34 genomes and aligned using gtdb-tk v.1.0.2 (Parks et al., 2018; Chaumeil et al., 2019). The concatenated alignment was refined using Gblock v.0.91b with the most gentle parameters and complete gap elimination option (Castresana, 2000). The final alignment contained 27,083 amino acids. A phylogenetic tree was constructed in RAxML v.8.2.12 (Stamatakis, 2014) with the PROTGAMMAILG model of amino acid substitutions. Local support values were 1000 rapid bootstrap replications.

### Genomic Analysis and Bioinformatics Methods

Carbohydrate-active enzyme (CAZyme) genes were searched with dbCAN2 (Zhang et al., 2018) using HMMER (Potter et al., 2018) with default parameters. For selected CAZymes, phylogenetic analysis was performed as follows: the top 5 BLAST homologs for each sequence and all proteins from the Swiss-Prot database (evidence at protein level only) affiliated to the corresponding family were aligned in Muscle (Edgar, 2004). Phylogenetic trees were constructed in RAxML v.8.2.12, as described above (*Phylogenetic analysis* section). Manual curation of protein function prediction was conducted using BlastP (McGinnis and Madden, 2004), HHpred (Zimmermann et al., 2018), HHMMER (phmmer and hmmscan) (Potter et al., 2018), and SUPERFAMILY 2 (Pandurangan et al., 2019). Secretion signal peptides and transmembrane domains were predicted using SignalP (Almagro Armenteros et al., 2019), Phobius (Käll et al., 2007), and TMHMM (Krogh et al., 2001). Search for orthologous gene clusters in *Thermococcus* sp. strains 2319x1 and 2319x1E was performed with OrthoVenn2 (Xu et al., 2019).

### Cultivation and Growth Experiments

*Thermococcus* sp. strain 2319x1E was isolated from the same *in situ* enrichment as the previously described *Thermococcus* sp. strain 2319x1 (Gavrilov et al., 2016). Cultivation as well as growth experiments were conducted in a strictly anaerobic modified Pfennig medium, consisting of 0.33 g l^–1^ MgCl_2_, CaCl_2_, KCl, NH_4_Cl and KH_2_PO_4_, 9 g l^–1^ NaCl, 2 g l^–1^ NaHCO_3_, 16 g l^–1^ Na_2_S ⋅ 9 H_2_O, 0.5 ml l^–1^ trace elements (Kevbrin and Zavarzin, 1992), 1 ml l^–1^ vitamin solution (Wolin et al., 1963), 1 g l^–1^ sulfur, 0.1 g l^–1^ yeast extract and 1 g l^–1^ of the respective growth substrate (beechwood xylan, Avicel^®^ cellulose, D-glucose, D-xylose). All growth experiments were performed at 85°C and pH 7.0 under a N_2_-atmosphere, either in 500 ml Schott bottles without shaking or in a 10 l bioreactor (Bioengineering, Switzerland) with constant stirring (100 rpm) and N_2_ infusion (0.5 l min^–1^). Growth was determined by counting cells in a Neubauer improved counting chamber (Brand, Germany) on a Zeiss Axioscope microscope (Zeiss, Germany). Cells that were further used for assays, ABPP experiments or proteomic analysis, were grown on the respective substrate for at least three consecutive transfers to the same fresh medium.

### Determination of Enzyme Activity in *Thermococcus* Cells

*Thermococcus* sp. strain 2319x1E cells were collected from grown cultures by centrifugation at up to 8,000 × *g* for 10 min at 25°C, washed two times with 1× PBS (8 g l^–1^ NaCl, 0.2 g l^–1^ KCl, 1.44 g l^–1^ Na_2_HPO4, 0.24 g l^–1^ KH_2_PO_4_, pH 7.0) and stored at –80°C. After thawing, the cell pellets were resuspended in 1× PBS pH 7.0 (2 ml per gram wet weight) and sonicated with a UP 200S sonicator (Hielscher Ultrasonics GmbH, Germany) for 3× 5 min (cycle 0.5, amplitude 50) followed by centrifugation at 12,000 × *g* for 30 min at room temperature to remove intact cells and cell debris. To separate the cytosolic protein fraction from the membrane protein fraction, ultracentrifugation of the crude protein extract was done (30 min, 100,000 × *g*, 4°C). To retrieve the respective membrane fraction, the pellet was resuspended in 1 × PBS pH 7.0 after collecting the cytosolic protein-containing supernatant. Protein concentrations were determined using the Bradford assay (Bio-Rad, United States) with bovine serum albumin (BSA) as standard (Bradford, 1976).

For determining glucosidase activities of *Thermococcus* sp. strain 2319x1E from either crude extracts or cytosolic and membrane fractions, the 3,5-dinitrosalicylic acid (DNSA) assay was applied (Miller, 1959). The enzyme assays were performed in 50 mM Tris-HCl pH 7.0 with a total reaction volume of 550 μl, containing 250 μl substrate solution in H_2_O (1% w/v for xylan and CMC, 0.04% w/v for xylobiose). The reactions were started by the addition of 7.5 μg of protein from the respective sample followed by incubation for 30 min at 85°C. The reaction mixture was cooled on ice for 5 min, mixed with 700 μl DNSA reagent (Miller, 1959) and subsequently incubated at 100°C for 10 min. The concentration of reducing sugars was then calculated from the absorption of the samples at 575 nm by using a D-xylose or D-glucose calibration curve. Background absorption of cell extracts and substrate solution was subtracted from the absorption of the samples. One unit of enzyme activity was defined as the amount of enzyme required to release 1 μmol D-xylose equivalents per minute. All absorbance measurements were done in 96-well plates using a Tecan infinite M200 plate reader (Tecan trading AG, Switzerland).

### Cloning of Glycoside Hydrolases for Protein Expression in *Escherichia coli*

The *Thermococcus* sp. strain 2319x1E genes *EGIDFPO_00674* (1800 nt) and *EGIDFPO_00532* (2196 nt) were amplified from gDNA extracted with the DNeasy Blood & Tissue Kit (Qiagen, Germany) using Q5^®^ polymerase (NEB, United States) and the following gene specific primers (Eurofins Genomics, Germany): for *EGIDFPOO_00674* 5′-GCGGCCCATGGGCTACCAGAAGTTTGGATATCATTTTCA TGC-3′ and 5′-ATTAGAATTCCTAGTGGTGGTGGTGGTGGT GAACCCTGGCCCTATGCTCGCACCATT-3′ (*Nco*I and *Eco*RI restriction sites underlined); for *EGIDFPOO_00532* 5′-CTGGTGCTAGCCTGATGCTTGTCTTTCCCGATTCCTTCC TCT-3′ and 5′-CTATCCTCGAGTCATAGCTCCGGAAGTCCAT ACTTTG-3′ (*Nhe*I and *Xho*I restriction sites underlined). PCR products were afterwards purified using the Wizard^®^ SV Gel and PCR clean-up kit (Promega, United States).

The genes were cloned into the pET-28b(+) vector (Novagen, United States) for subsequent expression, providing the recombinant proteins with a C-terminal (EGIDFPOO_00674) or N-terminal 6× His-tag. After restriction digest of the purified PCR products and the empty vector with the respective restriction enzymes (NEB), PCR product and vector were used in a molar ratio of 1:6 for *sticky end* ligation using T4 DNA ligase (NEB) at 16°C over night. *E. coli* DH5α cells (Novagene, United States) were transformed with the obtained constructs and the presence of successfully cloned genes was confirmed by colony PCR with the transformed cells as template and T7 promotor and terminator primers. The correctness of the sequence was confirmed by Sanger sequencing of both strands (Eurofins Genomics, Germany). The codon optimized gene (*E. coli*) *EGIDFPOO_00563* was synthesized and cloned into a pET28b(+) vector with N-terminal 6× His-tag (BioCat GmbH, Germany).

### Heterologous Expression and Purification of Glycoside Hydrolases

Recombinant expression of the glycoside hydrolases EGIDFPOO_00674, EGIDFPOO_00532 and EGIDFPOO_00563 was performed in *E. coli* Rosetta cells (Novagen, United States) which were transformed with the respective plasmids. A freshly inoculated 1 l culture in LB medium, supplemented with 50 μg ml^–1^ kanamycin and 50 μg ml^–1^ chloramphenicol, was grown to an OD_600_ of 0.4 at 37°C with constant shaking (180 rpm) upon subsequent induction of protein expression with 500 μM isopropyl-β-D-thiogalactopyranoside (IPTG). The cells were incubated at 18°C for a further 16 h, harvested by centrifugation (8,000 × *g* for 20 min at 4°C) and resuspended in 3–5 mL of buffer (50 mM Tris-HCl pH 7.0) per gram wet weight of the pellet for further protein purification. Cell lysis was performed by sonication as described above, followed by centrifugation at 12,000 × *g* for 45 min at 4°C. For further purification of EGIDFPOO_00674 or EGIDFPOO_00563, the cleared lysate was passed through a 0.45 μm filter, followed by an affinity purification using a 5 ml Ni-IDA column (GenScript, GenScript Biotech, United States) equilibrated with buffer A (50 mM Tris-HCl pH 7.8, 250 mM KCl, 10 mM imidazole). Protein elution was performed using a linear gradient of 10–350 mM imidazole in buffer A. The elution buffer was exchanged for storage buffer (50 mM Tris-HCl pH 8.0, 20 mM KCl, 5 mM MgCl_2_) using Amicon^®^ centrifugal filter devices (30 or 50 kDa cutoff, Merck, Germany). For long-time storage at –80°C, 50% (v/v) glycerol was added and the protein solution was flash frozen in liquid N_2_. EGIDFPOO_00532 was purified from inclusion bodies using a high pH-buffer purification strategy (Singh et al., 2015). The pellet obtained after sonication was washed with 50 mM Tris-HCl pH 8.0, taken up in 7.5 ml resuspension buffer (50 mM Tris-HCl pH 12.5, 2 M urea) per gram (wet weight) and subsequently incubated for 30 min at room temperature. The solubilized pellet was then centrifuged (8,000 × *g*, 10 min, 4°C) and the supernatant was diluted stepwise 1:20 into refolding buffer [50 mM Tris-HCl pH 8.0, 2 M urea, 10% (w/v) sucrose] on ice. Afterwards, a heat precipitation (20 min, 80°C) with subsequent centrifugation (8,000 × *g*, 10 min, 4°C) was performed. The obtained supernatant was directly used for further enzyme assays.

### Activity Assays With Recombinant Glycosidases

The activity of the heterologously expressed proteins EGIDFPOO_00532 and EGIDFPOO_00674 was determined using a discontinuous assay with the colorimetric nitrophenyl substrates PNPA, PNPX, PNPG, OPNG. Upon enzymatic cleavage of the nitrophenyl substrates, the absorbance of the free nitrophenol was measured at 400 nm in 96-well plates using a plate reader. Absorbance values of controls lacking the enzyme were subtracted from the absorbance values of the samples. Enzyme activity was calculated from a calibration curve with *para*-nitrophenol (PNP). The pH-dependent activity was measured at 348 nm in McIlvaine buffers with the respective pH values (McIlvaine, 1921) as described previously (Kallnik et al., 2014). Deacetylase activity of EGIDFPOO_00674 was measured in 50 mM Tris-HCl pH 8.0 using 2.5 μg of the purified enzyme and up to 8.8 mM PNPA in a total sample volume of 250 μl. α-amylase activity of EGIDFPOO_00674 was determined in 50 mM Tris-HCl pH 8.0 using the DNSA assay as described above, with 10 μg of the purified enzyme and 3 mM 6-*O*-α-maltosyl-β-cyclodextrin (MβCD) per 550 μl reaction volume. The β-glycosidase activity of EGIDFPOO_00532 was assessed in refolding buffer containing 25 μg of the purified enzyme and up to 26.7 mM of the respective PNP-substrate. Kinetics were determined at the optimal pH and temperature which was pH 8.0 and 100°C for both enzymes. To assess the thermostability of the enzymes, the PNP-substrate assay was performed as described above, but the enzymes were preincubated at 60, 80, and 100°C for up to 4 h. The glycogen phosphorylase activity of EGIDFPOO_00563 was assayed using a discontinuous enzyme assay; First, 500 μl samples containing 50 mM Tris-HCl pH 7, 20 μg purified EGIDFPOO_00563, 10 mM NaH_2_P0_4_ and 0.05% (w/v) substrate (glycogen, maltodextrin or starch) were incubated for 5 min at 100°C. Subsequently, the samples were stored on ice for 5 min. The amount of glucose 1-phosphate formed in the glycogen phosphorylase reaction was then determined by the indicator reaction, containing 4 U phosphoglucomutase (Sigma-Aldrich, United States), 3 U glucose-6-phosphate dehydrogenase (Roche, Switzerland), 10 mM MgCl_2_, and 0.5 mM NADP^+^ in 50 mM Tris-HCl pH 7 (total volume of 500 μl). The assay was incubated for 3 min at 30°C and the amount of glucose 1-phosphate was calculated from the absorption at 340 nm using a Specord 210^®^ photometer (Analytik Jena, Germany).

### Activity Inhibition With Activity-Based Probes

Protein solutions were diluted with their respective buffer to a final protein concentration of 0.02 mg protein ml^–1^ for native crude extract and 0.03 mg protein ml^–1^ for heterologously expressed purified protein. To examine activity inhibition upon covalent binding of the glycosidase probes JJB384 or JJB111, the samples (containing heterologously expressed proteins or *Thermococcus* sp. Strain 2319x1E crude extract) were incubated with 2 and 4 μM JJB384 or JJB111, respectively, for 1 h at 85 °C and subsequently used for activity determination assays with DNSA or nitrophenyl substrates.

### Sampling Cells for Proteomics Studies

Cells for proteomics studies were grown in Schott bottles as described above (Cultivation and Growth Experiments). For every carbon source under evaluation (beechwood xylan, Avicel^®^ cellulose, D-glucose, D-xylose), four biological replicates have been prepared. Therefore, 200 ml growth medium supplemented with the respective substrate were inoculated from a preculture which has been grown on that substrate for at least three consecutive transfers. Cells were harvested from cultures by centrifugation up to 8,000 × *g* for 20 min at 4°C after 10–12 h of growth during early mid-log phase. For storage at –80°C, the pellets were resuspended in 2 ml of the culture supernatant, transferred to fresh 15 ml Falcon tubes and centrifuged 6,500 × *g* for 10 min at 4°C. After removing the supernatant, the pellets were directly flash frozen in liquid N_2_.

### Protein Extraction for Full Proteome Studies and Activity-Based Protein Profiling Experiments

Cell pellets were resuspended in an adequate volume (approx. 2000–4000× less than the original culture volume) of 50 mM Na_2_HPO_4_ pH 8.0 supplemented with 1× MS-SAFE Protease and Phosphatase Inhibitor (Sigma-Aldrich, United States) and transferred to 1.5 ml reaction tubes. Cell lysis was performed by conducting a two-step sonication protocol. First, the cells were sonicated 7× 1 min in an ultrasonic bath filled with ice with short breaks in between to vortex the samples, followed by sonication with a Bioruptor UCD-200 (Diagenode, Belgium) using the following conditions: 1 min pulse and 30 s break in 10 cycles with the intensity set to “high” while cooled with ice. The extracts were cleared twice by centrifugation (21,000 × *g*, 20 min and 5 min, 4 °C) to remove cell debris and the protein concentration of the cleared extracts was determined by a modified Bradford assay with ROTI^®^Nanoquant (Carl Roth, Germany).

### Sample Preparation for Full Proteome Analysis

For each sample, 15 μg of total protein were subjected to a methanol-chloroform precipitation (Wessel and Flügge, 1984). The pellet was washed with methanol twice, dried on air and resuspended in 25 μl 8 M urea in 100 mM ABC, followed by reduction of disulfide bonds with 5 mM DTT in 100 mM ABC and incubation at 23°C for 30 min shaking at 1,000 rpm. Subsequently, alkylation of cysteine residues was done using 20 mM IAM in 100 mM ABC and incubation at 37°C for 30 min shaking in the dark at 1,000 rpm. The reaction was quenched by adding DTT to a final concentration of 25 mM. Afterwards, the proteins were digested with 500 ng Lys-C (FUJIFILM Wako Chemicals, Japan) dissolved in 100 mM ABC for 3 h at 37°C shaking at 1,000 rpm, followed by overnight digestion (∼16 h) with 500 ng trypsin (Thermo Scientific, United States) dissolved in 50 mM acetic acid at 37°C shaking at 1,000 rpm. Prior to sample clean-up for LC-MS/MS, formic acid (FA) was added to the samples to a final concentration of 5% (v/v).

In variation, the pellet of the samples from cells grown on xylan was washed once with 10% DMSO/10% ethanol and thrice with methanol after precipitation. Furthermore, the samples were dried in a vacuum concentrator (Eppendorf, Germany) after acidification and the peptides were dissolved in 0.5% (v/v) FA prior to desalting.

### *In vivo* Activity-Based Protein Profiling of Glycoside Hydrolases

All chemical probes were dissolved in DMSO. Labeling of active glycoside hydrolases was performed using a 2 ml liquid culture which was obtained by concentration of the growth culture (50× for samples analyzed on gel; 500× for samples dedicated for MS-based target identification) by centrifugation at up to 8,800 × *g* for 20 min at 25°C. For labeling, 2 μM of JJB384 or JJB111 were added to the liquid culture, followed by incubation at 78°C for 2 h shaking at 180 rpm. Afterwards, the cells were harvested by centrifugation at 8,800 × *g* for 10 min at 25°C. Protein extraction was performed as described above. For MS-based target identification, each sample was prepared in triplicates.

### Detection of Labeled Glycosidases by Western Blot Analysis

Labeled protein extracts (10–15 μg protein) were mixed with 4× lithium dodecyl sulfate (LDS) gel loading dye [423 mM Tris-HCl, 563 mM Tris base, 8% (w/v) LDS, 40% (w/v) glycerol, 2 mM EDTA, 0.075% (w/v) SERVA Blue G250; freshly supplemented with 100 mM DTT], incubated at 70°C for 15 min and separated by denaturing polyacrylamide gel electrophoresis on 11% Bis-Tris resolving gels. The separated proteins were transferred on a PVDF membrane (Merck, United States) using a tank blot setup (Bio-Rad, United States) and the membrane was washed thrice with TBS-T [20 mM Tris base pH 7.5, 150 mM NaCl, 0.2% (w/v) Tween20]. Blocking with 3% BSA (w/v) in TBS-T was done overnight at 4°C, followed by incubation with a streptavidin-horse radish peroxidase (HRP) conjugate (Sigma-Aldrich, United States) directly added into the blocking solution (1:25,000) for 2.5 h at room temperature. The membrane was washed 6× with TBS-T and the labeled proteins were detected by enhanced chemiluminescence (ECL) with a mix of the SuperSignal^®^ West Pico Chemiluminescent Substrate and the SuperSignal^®^ West Femto Maximum Sensitivity Substrate (4:1; Thermo Scientific, United States) using an Amersham Imager 600 (GE Healthcare Life Sciences, Germany).

### Enrichment of Labeled Glycoside Hydrolases and Sample Preparation for Mass Spectrometry-Based Proteomics

Prior to affinity enrichment, the protein solution (225 μg protein in a final volume of 500 μl 50 mM Na_2_HPO_4_ pH 8.0 supplemented with 1× MS-SAFE Protease and Phosphatase Inhibitor) was cleaned-up by precipitation with a 4× volume of methanol overnight at –20°C. The proteins were collected by centrifugation at 21,000 × *g* for 10 min at 4°C and washed with a 2× volume of methanol. The air-dried pellets were dissolved in 850 μl 2% (w/v) SDS in 1× PBS (155 mM NaCl, 3 mM Na_2_HPO_4_, 1.06 mM KH_2_PO_4_, pH 7.4) by incubation at 37°C with agitation at 1,000 rpm and diluted with 1× PBS pH 7.4 to a final concentration of 0.2% (w/v) SDS. The resulting protein solutions were incubated with avidin agarose beads (Thermo Scientific, United States), equilibrated in the same buffer, for 1 h at room temperature while gently tumbling. The beads were collected by centrifugation (400 × *g*, 5 min), washed 5× with 1% (w/v) SDS in MS-quality water and finally 4× with pure MS-quality water. Afterwards, tryptic on-bead-digestion was conducted. Thereto, the beads were taken up in 100 μl 0.8 M urea in 50 mM ABC buffer. Reduction of disulphide bonds with 10 mM DTT in 50 mM ABC was done by incubation at room temperature for 1 h shaking at 1,500 rpm, followed by alkylation of cysteine residues with 25 mM IAM in 50 mM ABC and incubation at room temperature for 1 h in the dark with shaking at 1,500 rpm. The reaction was quenched by increasing the DTT concentration to 35 mM and further 10 min incubation. For digestion of proteins, 10 μl of a 100 ng μl^–1^ trypsin stock solution in 50 mM acetic acid were added and the samples were incubated at 37°C for 16 h with shaking at 1,250 rpm. The beads were collected by centrifugation at 3,000 × *g* for 5 min and FA was added to the recovered supernatants to a final concentration of 5% (v/v). The beads were washed with 50 μl 1% (v/v) FA at room temperature for 15 min shaking at 1,500 rpm and collected by centrifugation at 3,000 × *g* for 5 min. Both supernatants were combined and subsequently cleared by passing over home-made tips containing two disks of glass microfiber (GE Healthcare, Life Sciences, Germany; poresize 1.2 μm; thickness 0.26 mm).

### Sample Clean-Up for LC–MS/MS

Peptides were desalted on home-made C_18_ StageTips (Rappsilber et al., 2007) containing two layers of an octadecyl silica membrane (3M, United States). All centrifugation steps were carried out at room temperature. The StageTips were first activated and equilibrated by passing 50 μl of methanol (600 × *g*, 2 min), 80% (v/v) acetonitrile (ACN) with 0.5% (v/v) FA (600 × *g*, 2 min) and 0.5% (v/v) FA (800 × *g*, 3 min) over the tips. Next, the tryptic digests were passed over the tips (800 × *g*, 3–4 min). The flow through was collected and applied a second time (same settings). The immobilized peptides were then washed with 50 μl and 25 μl 0.5% (v/v) FA (800 × *g*, 3 min). Bound peptides were eluted from the StageTips by an application of two rounds of 25 μl 80% (v/v) ACN with 0.5% (v/v) FA (600 × *g*, 2 min). After elution from the StageTips, the peptide samples were dried using a vacuum concentrator (Eppendorf, Germany) and the peptides were dissolved in 15 μl 0.1% (v/v) FA prior to analysis by MS.

### LC-MS/MS Analysis

Experiments were performed on an Orbitrap Elite or Orbitrap Fusion Lumos mass spectrometer (Thermo Fischer Scientific, United States) that were coupled to an EASY-nLC 1000 or 1200 liquid chromatography (LC) system (Thermo Fischer scientific, United States). The LCs were operated in the one-column mode. The analytical column was a fused silica capillary (inner diameter 75 μm × 36 or 46 cm) with an integrated PicoFrit emitter (New Objective, United States) packed in-house with Reprosil-Pur 120 C18-AQ 1.9 μm (Dr. Maisch, Germany). The analytical column was encased by a column oven (Sonation, Germany) and attached to a nanospray flex ion source (Thermo Fischer scientific, United States). The column oven temperature was adjusted to 45 or 50°C during data acquisition. The LC was equipped with two mobile phases: solvent A (0.1% FA, in water) and solvent B (nLC 1000: 0.1% FA in ACN; nLC 1200: 0.1% FA, 20% H_2_O, in ACN). All solvents were of UHPLC (ultra-high-performance liquid chromatography) grade (Honeywell, Germany). Peptides were directly loaded onto the analytical column with a maximum flow rate that would not exceed the set pressure limit of 980 bar (usually around 0.5 – 0.8 μl min^–1^). Peptide solutions were subsequently separated on the analytical column using different gradients (for details see Supplementary Data Sheet 2).

The mass spectrometers were operated using Xcalibur software (Elite: v2.2 SP1.48; Lumos: v4.3.7.3.11). The mass spectrometers were set in the positive ion mode. Precursor ion scanning (MS^1^) was performed in the Orbitrap analyzer (FTMS; Fourier Transform Mass Spectrometry with the internal lock mass option turned on (lock mass was 445.120025 *m*/*z*, polysiloxane) (Olsen et al., 2005). MS^2^ Product ion spectra were recorded only from ions with a charge higher than +1 and in a data dependent fashion in the ion trap mass spectrometry. All relevant MS settings (Resolution, scan range, AGC, ion acquisition time, charge states isolation window, fragmentation type and details, cycle time, number of scans performed, and various other settings) for the individual experiments can be found in Supplementary Data Sheet 2.

### Peptide and Protein Identification Using MaxQuant and Perseus

RAW spectra were submitted to an Andromeda search (Cox et al., 2011) in MaxQuant (v.1.6.2.6) using the default settings (Cox and Mann, 2008). Label-free quantification (LFQ) (Cox et al., 2014) and match between runs was activated. MS/MS spectra data were searched against the self-constructed TspE_proteome_AA_new.fasta file (2182 entries; see above for reference). All searches included a contaminants database (as implemented in MaxQuant, 246 sequences). The contaminants database contains known MS contaminants and was included to estimate the level of contamination. Andromeda searches allowed oxidation of methionine residues (16 Da) and acetylation of the protein N-terminus (42 Da) as dynamic modifications while carbamidomethylation of cysteine residues (57 Da, alkylation with IAM) was selected as static modification. Enzyme specificity was set to “Trypsin/P.” The instrument type in Andromeda searches was set to Orbitrap and the precursor mass tolerance was set to ±20 ppm (first search) and ±4.5 ppm (main search). The MS/MS match tolerance was set to ±20 ppm. The peptide spectrum match FDR and the protein FDR were set to 0.01 (based on target-decoy approach). Minimum peptide length was 7 amino acids. For protein quantification, unique and razor peptides were allowed. In addition to unmodified peptides, modified peptides with dynamic modifications were allowed for quantification. The minimum score for modified peptides was set to 40.

Further data analysis and filtering of the MaxQuant output was done in Perseus (v.1.6.2.1) (Tyanova et al., 2016). LFQ intensities were loaded into the matrix from the proteinGroups.txt file and potential contaminants as well as reverse hits from the reverse database and hits only identified based on peptides with a modification site were removed. For all MS-based proteomics experiments, biological replicates were combined into categorical groups to allow comparison of the different treatment groups and the data was transformed to the log_2_-scale. For the full proteome analysis, the data was filtered to keep only hits with a valid LFQ intensity for at least three out of four replicates for samples from cells grown on xylan. For the identification of glycoside hydrolases enriched with JJB384, the data was separately filtered for the two sugar substrates under investigation. Only hits with a valid LFQ intensity for a minimum of two out of three of the probe-labeled sample replicates were retained for further analysis. Prior to quantification, missing values were imputed from a normal distribution (width 0.3, down shift 1.8). Comparison of normalized protein group quantities (relative quantification) between different MS runs was solely based on the LFQ intensities as calculated by MaxQuant (MaxLFQ algorithm) (Cox et al., 2014). Briefly, label-free protein quantification was switched on and unique and razor peptides were considered for quantification with a minimum ratio count of 2. Retention times were recalibrated based on the built-in non-linear time-rescaling algorithm. MS/MS identifications were transferred between LC-MS/MS runs with the “Match between runs” option in which the match time window was set to 0.7 min and the alignment time window to 20 min. The quantification was based on the “value at maximum” of the extracted ion current. At least two quantitation events were required for a quantifiable protein. The log_2_-fold enrichment of protein groups for samples from cells grown on xylan was calculated based on the mean LFQ intensity compared to the samples from cells grown on any of the other sugars. Statistical significance was calculated based on a two-sided Student’s *t*-test. Full MS data for the comparative full proteome analysis can be found in Supplementary Table 1. The log_2_-fold enrichment of protein groups with JJB384 was calculated based on the mean LFQ intensity compared to the DMSO control. Protein groups with a negative fold enrichment were excluded from further analysis. Full MS data for the ABPP with JJB384 can be found in Supplementary Table 2.

## Results

### *Thermococcus* sp. Strain 2319x1E Genome Analysis

**FIGURE 2:**
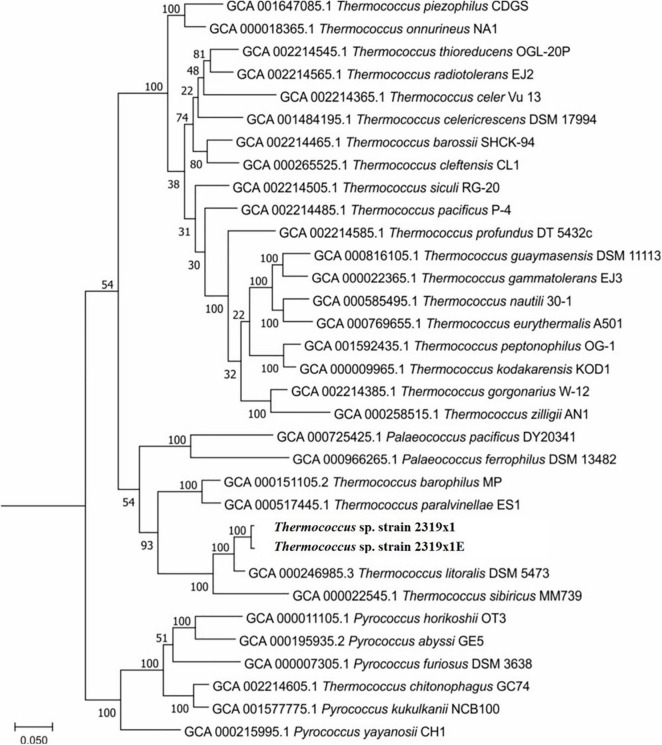
Phylogenetic position of the novel *Thermococcus* sp. strain 2319x1E in comparison with all available cultivated genomes of *Thermococcus*, *Pyrococcus*, and *Palaecoccus* species. Tree construction was done in RAxML v. 8.2.12 with the PROTGAMMAILG model of amino acid substitutions.

*Thermococcus* sp. strain 2319x1E was isolated from the same enrichment culture as *Thermococcus* sp. strain 2319x1 (Gavrilov et al., 2016). The fully assembled genome of *Thermococcus* sp. strain 2319x1E consists of a single chromosome with the size of 1,989,851 bp and an average GC content of 44.39%. The sequence is deposited in the NCBI genome database under accession number LR778300. The average nucleotide identity (ANI) to the genome of *Thermococcus* sp. strain 2319x1 is 97.86%, thus being above the species level (95%) according to Goris et al. (2007), whereas the ANI to *Thermococcus litoralis*, which is considered the closest validly published *Thermococcus* species described, is only 90.01% and to the more distantly related *Thermococcus kodakarensis* 71.97%. The phylogenetic position of both *Thermococcus* isolates was determined using phylogenetic tree construction based on the “ar122” set of conserved proteins (Figure 2).

The predicted number of protein-coding sequences (CDS) in *Thermococcus* sp. strain 2319x1E is 2,195, and therefore is in a similar range as the 2319x1 genome (2,192 CDS). The comparison of both genomes revealed 1,886 single-copy orthologous gene clusters found in both strains and 266 and 360 unique genes in the genome of 2319x1E and 2319x1, respectively. Altogether, these numbers indicate that the two *Thermococcus* strains are relatively closely related to each other. With regard to enzymes involved in xylan degradation, the CAZymes present in both strains were compared. The *Thermococcus* sp. strain 2319x1E genome comprises 42 genes which putatively encode for CAZymes (Lombard et al., 2014), with 26 of them being predicted to encode for glycosyl transferases (GT), 13 for glycosyl hydrolases (GH) and 3 for carbohydrate esterases (CE) (Table 1). Remarkably, the genes encoding the unique multifunctional multidomain glycosidase described for the *Thermococcus* sp. strain 2319x1 isolate (Gavrilov et al., 2016) and 4 additional GHs, as well as 8 GTs are not present in the genome of the new isolate 2319x1E. Correspondingly, in the *Thermococcus* sp. strain 2319x1, genes encoding 9 GTs and 2 GHs (EGIDFPOO_01323 and EGIDFPOO_01324) that are present in strain 2319x1E are missing (Supplementary Table 1 in Supplementary Data Sheet 1).

**TABLE 1:** CAZymes identified in the *Thermococcus* sp. strain 2319x1E genome.

CAZy domain	Predicted function	Gene	Secretion signal	Transmembrane domain
**Glycosyl hydrolases**				
GH13	α-1,4-glucan:maltose-1-phosphate maltosyltransferase	*EGIDFPOO_00018*		(+)
GH130	β-1,4 -mannooligosaccharide phosphorylase	*EGIDFPOO_00274*		
GH57	α-amylase/4-α-gluconotransferase	*EGIDFPOO_00375*		
GH1	β-glucosidase	*EGIDFPOO_00532*		
GH57		*EGIDFPOO_00534*		
GH57		*EGIDFPOO_00674*		
GH122	α-glucosidase	*EGIDFPOO_00753*		
GH57	1,4-α-branching enzyme	*EGIDFPOO_01266*		
	Amylo- α -1,6-glucosidase	*EGIDFPOO_01323*		
GH1	β-glucosidase	*EGIDFPOO_01324*		
GH57		*EGIDFPOO_01845*	Sec/SPI	+
GH13	α-amylase/neopullulanase	*EGIDFPOO_01849*		
GH13	α-amylase/neopullulanase	*EGIDFPOO_01993*	Sec/SPII	+
**Carbohydrate esterases**				
CE10		*EGIDFPOO_00955*		
CE10		*EGIDFPOO_01219*		
CE1	Putative carbohydrate esterase	*EGIDFPOO_01302*	Sec/SPII	
**Glycosyl transferases**				
GT2	Undecaprenyl-phosphate 4-deoxy-4-formamido-L-arabinose transferase	*EGIDFPOO_00012*		++
GT55		*EGIDFPOO_00184*		
GT66		*EGIDFPOO_00197*	Sec/SPI	++
GT39		*EGIDFPOO_00332*	Sec/SPI	++
GT66		*EGIDFPOO_00336*		++
GT2	Undecaprenyl-phosphate mannosyltransferase	*EGIDFPOO_00399*		
GT35	Glycogen phosphorylase	*EGIDFPOO_00563*		
GT2	Phosphoglycolate phosphatase	*EGIDFPOO_00612*		
GT4	*N*-acetyl-α-D-glucosaminyl L-malate synthase	*EGIDFPOO_01181*		
GT4	*N*-acetyl-α-D-glucosaminyl L-malate synthase	*EGIDFPOO_01440*		+
GT81	Glucosylglycerate synthase	*EGIDFPOO_01674*		
GT5	Glycogen synthase	*EGIDFPOO_01851*		
GT2	Undecaprenyl-phosphate 4-deoxy-4-formamido-L-arabinose transferase	*EGIDFPOO_01900*		++
GT66		*EGIDFPOO_02109*		++
GT2	Undecaprenyl-phosphate 4-deoxy-4-formamido-L-arabinose transferase	*EGIDFPOO_02112*		++
GT4	D-inositol-3-phosphate glycosyltransferase	*EGIDFPOO_02119*		
GT4	D-inositol-3-phosphate glycosyltransferase	*EGIDFPOO_02121*		
GT4	D-inositol-3-phosphate glycosyltransferase	*EGIDFPOO_02122*		
GT4	Spore coat protein SA	*EGIDFPOO_02135*		
GT4		*EGIDFPOO_02136*		
GT4	D-inositol-3-phosphate glycosyltransferase	*EGIDFPOO_02137*		
GT2		*EGIDFPOO_02138*		++
GT2		*EGIDFPOO_02139*		+
GT4	D-inositol-3-phosphate glycosyltransferase	*EGIDFPOO_02196*		
GT2		*EGIDFPOO_02198*		+
GT55		*EGIDFPOO_02209*		

*The table shows all putative CAZymes with their corresponding predicted function, predicted secretion signal and predicted occurrence of transmembrane domains. Sec/SPI or Sec/SPII stands for Sec-type signal peptides or lipoprotein signal peptides, respectively; + indicates the presence of one predicted transmembrane (TM) domain; ++ indicates at least two predicted TM domains; (+) indicates that a TM domain was only predicted with Phobius, but not with TMHMM.*

Of the CAZymes identified in *Thermococcus* sp. strain 2319x1E, two putative GHs encoded by the genes EGIDFPOO_01845 and EGIDFPOO_01993 were predicted to be extracellular (Table 1), although it should be noted that the reliable prediction of archaeal secretion signals remains challenging (Bagos et al., 2009; Szabo and Pohlschroder, 2012). In addition, 11 putative GTs were predicted to be membrane-associated (Table 1) due to the presence of one or more predicted transmembrane domains. Among the 12 putative GHs, 7 are most likely involved in the hydrolysis of α-linked oligo- and polymers such as glycogen, starch, pullulan and maltooligosaccharides, as determined by BLAST against the Swiss-Prot database as well as phylogenetic analyses for some families (GH13 and GH57; Supplementary Figures 1, 2 in Supplementary Data Sheet 1). In addition, three enzymes were annotated as β-specific hydrolases: EGIDFPOO_00274 is predicted to act as a β-1,4-mannooligosaccharide phosphorylase (GH130), while the two GHs EGIDFPOO_00532 and EGIDFPOO_01324 are putative β-glucosidases (GH1). However, no homologs of enzymes known to be involved in xylan degradation, such as endo-xylanases, β-xylosidases, α-arabinofuranosidases, α-glucuronidases or acetylxylan esterases, were identified.

### Growth Characteristics of *Thermococcus* sp. Strain 2319x1E

Cells of *Thermococcus* sp. strain 2319x1E show a morphology typical for *Thermococcales* as they appear as irregularly shaped cocci with a size of 1–2 μm in diameter. This strain is an obligate anaerobe and grows optimal at 85°C and a pH of 7.0. When cultivated on xylan in batch mode, the generation time was 3.4 h (2.9 h for D-xylose, 3.7 h for Avicel^®^ cellulose, 3.2 h for D-glucose) and the final growth yield ranged 1–1.5 × 10^7^ cells ml^–1^ for all substrates (see Supplementary Figure 3 in Supplementary Data Sheet 1). Notably, cell lysis was observed after 20 h of cultivation by DAPI staining, indicating a short stationary phase when the cells were grown in a closed bottle without stirring and gas exchange.

### Proof of Native Xylanolytic Activity

**FIGURE 3:**
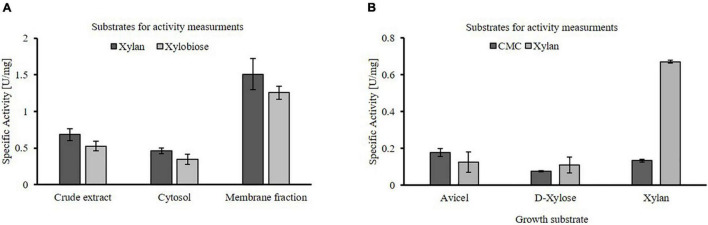
Hydrolytic activity of *Thermococcus* sp. strain 2319x1E cell fractions after growth on different substrates. **(A)** Specific hydrolytic activity of distinct protein fractions from cells grown on beechwood xylan using xylan or xylobiose as substrate, determined by monitoring the increase of reducing sugars via the DNSA assay. The activity in crude extracts, the soluble cytosolic and the membrane fraction is shown. **(B)** Specific enzymatic activity in crude extracts of cells grown on either Avicel^®^ cellulose, D-xylose or beechwood xylan using xylan or CMC as substrate. The enzymatic activity was determined after 30 min of incubation at 85°C using the DNSA assay.

*Thermococcus* sp. strain 2319x1E crude extracts as well as the isolated cytosolic and membrane fractions obtained from cells grown on xylan possess hydrolytic activity on xylan and xylobiose, similar to strain 2319x1 (Figure 3A). The specific xylanolytic activity of the membrane fraction was roughly three times higher than for the cytosolic fraction, thus indicating the presence of membrane-bound hydrolase activity. Furthermore, the hydrolytic activity in crude extracts of cells grown on xylan, D-xylose and Avicel^®^ cellulose was compared using either xylan or CMC as substrate (Figure 3B). Whereas a higher xylanolytic activity is clearly linked to growth on xylan, only a slight effect of the growth substrate could be observed for the glucosidase activity on CMC compared to xylan. Of note, no activity was observed in the culture supernatant using the DNSA assay despite long incubation times. Additional efforts to concentrate the supernatant by means of centrifugation or filtration failed due to the viscosity of the residual xylan.

### Changes in Protein Abundance in Response to Different Sugars

**FIGURE 4:**
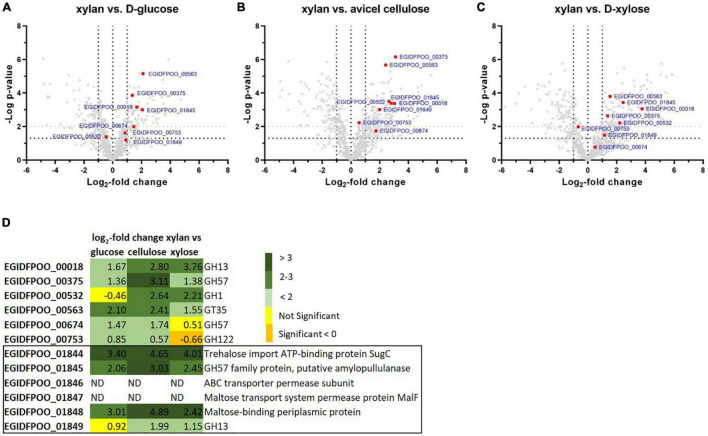
Differentially abundant proteins in *Thermococcus* sp. strain 2319x1E cells grown on different carbon sources. The protein abundance profile of cells grown on beechwood xylan was compared with that of cells grown either on D-glucose **(A)**, Avicel^®^ cellulose **(B)** or D-xylose **(C)**. The volcano plots show the *p*-value against the log_2_-fold change in protein abundance. Statistical significance was calculated based on a two-sample Student‘s *t*-test. Predicted CAZymes are depicted as red squares. Each treatment group comprised four biological replicates. **(D)** Table of differentially abundant CAZymes or selected sugar transport proteins with their respective log_2_-fold change and annotation. Yellow fields indicate non-significantly regulated proteins, orange fields indicate significantly downregulated proteins (*p* < 0.05), whereas green fields indicate significantly upregulated proteins (*p* < 0.05) with a log_2_-fold change > 1 (pale green: 1–2; moderate green: 2–3; dark green: >3). Proteins that were absent in the filtered data are displayed as ND (not detected). The two GHs EGIDFPOO_01845 and EGIDFPOO_01849 belong to a gene cluster encoding further sugar transport proteins that is boxed.

In order to first identify proteins from *Thermococcus* sp. strain 2319x1E that are regulated in response to the offered carbon source, we used a comparative proteomics approach. *Thermococcus* sp. strain 2319x1E cells were grown on either D-xylose, D-glucose, Avicel^®^ cellulose or beechwood xylan with subsequent MS-based full proteome analysis to identify differentially abundant glycoside hydrolases based on LFQ (Figure 4 and Supplementary Table 1).

Statistical evaluation of the identified proteins showed the strong alteration of protein abundances upon growth of *Thermococcus* sp. strain 2319x1E on beechwood xylan compared to D-glucose (Figure 4A), Avicel^®^ cellulose (Figure 4B), and D-xylose (Figure 4C). The abundance of 107 proteins was significantly upregulated with a log_2_-fold change > 1 in the proteome of xylan-grown cells compared to cells grown on D-glucose (200 proteins compared to cellulose, 208 proteins compared to D-xylose) whereas 90 proteins were significantly downregulated with a log_2_-fold change < -1 (114 proteins compared to cellulose and D-xylose). Among the proteins that showed a differential abundance in response to growth on the different sugars, we found several CAZymes (for overview see Figure 4D and Supplementary Table 2 in Supplementary Data Sheet 1). The α-specific CAZymes EGIDFPOO_00018 (GH13), EGIDFPOO_00375 (GH57), EGIDFPOO_00563 (GT35), EGIDFPOO_00674 (GH57) EGIDFPOO_01845 (GH57), and EGIDFPOO_01849 (GH13) were all found to be more abundant in cells grown on xylan compared to the other tested sugars, although this difference was not significant for EGIDFPOO_00674 compared to growth on D-xylose as well as for EGIDFPOO_01849 compared to growth on D-glucose. EGIDFPOO_00532, a predicted β-glucosidase, was less abundant in cells grown on xylan when compared to cells grown on D-glucose, but more abundant compared to cells grown on Avicel^®^ cellulose or D-xylose. Moreover, the GH122 family hydrolase EGIDFPOO_00753 was slightly upregulated in xylan-grown cells compared to D-glucose and Avicel^®^ cellulose, but slightly downregulated compared to D-xylose. Noteworthy, the genes for the two hydrolases *EGIDFPOO_01845* (GH57) and *EGIDFPOO_01849* (GH13) are in close genetic neighborhood to a predicted ATP-binding cassette (ABC) transporter comprising the genes EGIDFPOO_01846 and EGIDFPOO_01847 (both permeases), as well as to EGIDFPOO_01844 and EGIDFPOO_01848, which are predicted to encode for an ATP binding protein and a putative sugar binding protein, respectively. These proteins are homologous to subunits of an ABC transporter for maltose, trehalose and probably a number of other mono- and disaccharides (Greller et al., 1999). The abundance of this gene cluster is upregulated in cells grown on xylan compared to cells grown on Avicel^®^ cellulose, D-glucose or D-xylose. However, the putatively membrane bound sugar transport proteins EGIDFPOO_01846 and EGIDFPOO_01847 were removed from the data due to the filtering criteria applied (see methods; Supplementary Table 1). The log_2_-fold changes for all candidate proteins are reported (Figure 4D).

### Identification of Active Enzymes Involved in Xylan Degradation by Activity-Based Protein Profiling

**FIGURE 5:**
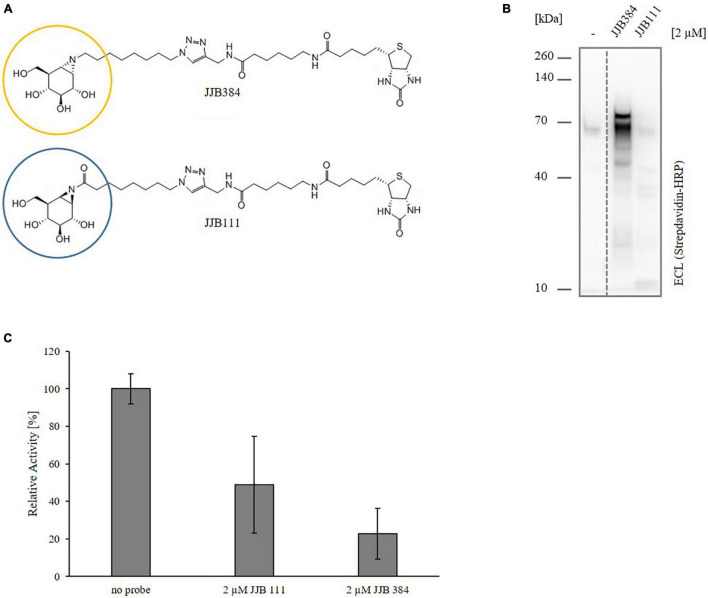
ABPP of *Thermococcus* sp. strain 2319x1E with glycosidase-selective probes. **(A)** Chemical structure of the two employed glycosidase-selective ABPs JJB384 and JJB111. The circles indicate the cyclophellitol aziridine moieties (yellow, α-configured moiety of JJB384; blue, β-configured moiety of JJB111) which function as warheads covalently binding to the target proteins. **(B)** Western blot analysis of the in vivo profiled *Thermococcus* sp. strain 2319x1E proteome of cells grown on xylan after separation via Bis-Tris gel electrophoresis and detection with a streptavidine HRP conjugate. The cells were incubated with 2 μM of either of the two ABPs or with DMSO as control for 2 h at 78°C with constant shaking. **(C)** Xylanolytic activity of *Thermococcus* sp. strain 2319x1E cell extracts (20 μg protein) after incubation in presence or absence of 2 μM of the respective probe for 2 h at 78°C. Inhibition of xylanolytic activity was determined in triplicates.

To shift the focus on the detection of active glycosidases involved in xylan degradation in *Thermococcus* sp. strain 2319x1E, we screened the two well-established biotin-tagged cyclophellitol aziridine-based glycosidase probes JJB384 and JJB111 (Figure 5A) for their ability to label active enzymes *in vivo* upon growth on xylan. Thereto, *Thermococcus* sp. strain 2319x1E cells grown on xylan were incubated with 2 μM of the respective probe for 2 h, followed by the detection of labeled proteins via Western blot analysis (Figure 5B). This revealed a distinctive band pattern for the samples labeled with JJB384 with two prominent bands at a MW around 70 kDa. While the upper band is absent in the DMSO control, there is a band in the control sample at about the same height as the lower band in the labeled sample, but considerably less pronounced. In contrast, only faint bands are visible for the labeling with JJB111. Furthermore, the inhibitory effect of both ABPs on the xylanolytic activity was tested. Crude extract of *Thermococcus* sp. strain 2319x1E cells grown on xylan was incubated under the conditions previously described for the *in vivo* labeling, after which activity determination was performed via the DNSA assay. Despite only a faint labeling was obtained from the cells incubated with JJB111 in the Western blot analysis, the hydrolytic activity of the crude extract on xylan was decreased to 49% by preincubation with the probe. However, this effect was distinctly stronger for JJB384, with a remaining activity of about 23% (Figure 5C). Consequently, JJB384 was selected for further identification of target enzymes by MS-based proteomics.

**FIGURE 6:**
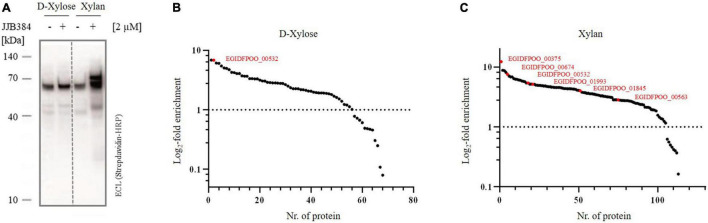
CAZymes identified by ABPP with JJB384. **(A)** Western blot analysis of the *in vivo* profiled *Thermococcus* sp. strain 2319x1E proteome of cells grown on either D-xylose or xylan after separation via Bis-Tris gel electrophoresis and detection with a streptavidine-HRP conjugate. The cells were incubated with 2 μM JJB384 for 2 h at 78°C with constant shaking. **(B,C)** Log_2_-fold changes for all proteins enriched with JJB384 in cells grown on D-xylose **(B)** or xylan **(C)** compared to the respective DMSO-controls lacking the probe. Predicted CAZymes are depicted in red. Each treatment group comprised three biological replicates.

To confirm that the band pattern obtained by labeling with JJB384 is specific for cells grown on xylan, a comparative *in vivo* ABPP of *Thermococcus* sp. strain 2319x1E grown on either D-xylose or beechwood xylan was conducted (Figure 6A). The Western blot analysis shows a differential labeling pattern of proteins from cells grown on the different carbon sources. In contrast to the labeling of cells grown on xylan, no differences in the band pattern between labeled cells and the control could be observed for cells grown on D-xylose. Thus, proteins that were not labeled with JJB384 after growth of cells on D-xylose show specific activity upon growth of cells on xylan and therefore may play a role in xylan degradation.

For identification of the JJB384 probe-labeled proteins, an affinity enrichment with subsequent tryptic on-bead digestion and LC-MS/MS analysis was performed using protein extracts from cells grown on D-xylose and beechwood xylan (Figures 6B,C and Supplementary Table 2). The initial data were filtered (see methods) to only keep protein groups that were identified in at least two out of three biological replicates of probe-labeled samples per carbon source. For the xylan samples, 122 protein groups were retained, of which 113 protein groups were enriched with the probe compared to the DMSO controls, whereas 68 out of 69 retained protein groups were enriched with JJB384 for the D-xylose samples. Among the xylan samples, 5 GHs (EGIDFPOO_00375, EGIDFPOO_00532, EGIDFPOO_00674, EGIDFPOO_01845, EGIDFPOO_01993) and 1 GT (EGIDFPOO_00563) were enriched with JJB384 (Figure 6C). Of these, only EGIDFPOO_01993 was not reported in the comparative full proteome analysis, as this protein did not pass the filtering criteria and was therefore removed from the initial dataset (see methods, Supplementary Table 1).

We next had a closer look at these active enzymes that likely participate in xylan degradation. EGIDFPOO_00532 is the only β-specific GH among the identified proteins and is closely related to β-glucanases and β-galactosidases from other thermophilic archaea (Supplementary Figure 4 in Supplementary Data Sheet 1 and Supplementary Table 3 in Supplementary Data Sheet 1). Notably, it is not only enriched in the xylan samples, but is also the only CAZyme enriched in the D-xylose samples (Figure 6B). The respective log_2_-fold changes for all identified CAZymes are depicted in Table 2. Several α-specific CAZymes were thus only enriched in the xylan samples. The highest enrichment was observed for EGIDFPOO_00375 from the GH57 family (Supplementary Figure 2 in Supplementary Data Sheet 1) that is homologous to the characterized pullulan hydrolase TK-PUL from *T. kodakarensis* (Ahmad et al., 2014), 4-α-glucanotransferase from *T. litoralis* (Jeon et al., 1997) and α-amylase from *P. furiosus* (Laderman et al., 1993). These enzymes have been reported to hydrolyze multiple α-linked polysaccharides, including starch, glycogen, dextrin, amylose, amylopectin, and different cyclodextrins. According to phylogenetic analysis and BLAST with biochemically characterized enzymes, other enriched hydrolase candidates were predicted to function as amylopullulanase (EGIDFPOO_001845, Supplementary Figure 2 in Supplementary Data Sheet 1) or glucan-branching enzyme (EGIDFPOO_01993, Supplementary Figure 1 in Supplementary Data Sheet 1) based on PFAM analysis and sequence homology to biochemically described enzymes (Han et al., 2013; Sun et al., 2015) and should therefore also be specific for hydrolysis of α-linkages in polymers. EGIDFPOO_00563 is highly similar to the GT35 family maltodextrin phosphorylase from *T. litoralis* (97% sequence identity) (Xavier et al., 1999). Notably xylanase activity was reported for the characterized homolog from *T. zilligii* (76% sequence identity) (Uhl and Daniel, 1999), which was, however, later questioned and the respective protein is now annotated as a maltodextrin-phosphorylase in the PDB database (Rolland et al., 2002). EGIDFPOO_00674 is another putative α-specific glycosidase enriched in the xylan samples that belongs to the highly diverse GH57 family and, like EGIDFPOO_00375 and EGIDFPOO_01845, forms an isolated cluster within the phylogenetic tree (Supplementary Figure 2 in Supplementary Data Sheet 1). Unlike the other GH57 family proteins found with the ABPP approach, EGIDFPOO_00674 was not assigned a specific function by automated proteome annotation in Swiss-Prot (Gattiker et al., 2003) and was therefore selected for biochemical characterization along with the putative β-glucosidase EGIDFPOO_00532. The second predicted β-glucosidase EGIDFPOO_01324 (Table 1) was neither identified with the comparative full proteome survey nor with the ABPP approach.

**TABLE 2:** Overview of CAZymes enriched from *Thermococcus* sp. strain 2319x1E cultures grown on xylan or D-xylose using the α-selective probe JJB384.

Growth substrate	Gene	Protein family and predicted function	MW [kDa]	Log_2_-fold change	Domain architecture (PFAM)
Xylan	*EGIDFPOO_00375*	GH57, **α (1–4)** amylase/4-α-glucanotransferase	47.7	12.27	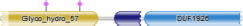
	*EGIDFPOO_00674*	GH57, **α**, putative GH57 family enzyme	70.6	7.57	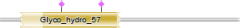
	*EGIDFPOO_00532*	GH1, **β (1–4)** glucosidase	49.3	5.39	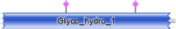
	*EGIDFPOO_01993*	GH13, **α (s1–4)** glucan branching enzyme	82.7	5.16	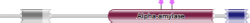
	*EGIDFPOO_01845*	GH57, **α (1–4/6)** amylopullulanase	125.2	4.01	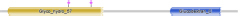
	*EGIDFPOO_00563*	GT35, **α (1–4)** glycogen phosphorylase	96.8	2.82	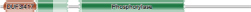
D-Xylose	*EGIDFPOO_00532*	GT1, **β (1–4)** glucosidase	49.3	6.82	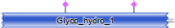

*Log_2_-fold changes indicate the difference in LFQ intensity between the labeled samples and the DMSO controls. The respective domain architectures, including predicted active site residues, were obtained with the HMMER function phmmer.*

In summary, of the 13 GHs identified in the genome of *Thermococcus* sp. strain 2319x1E (Table 1), 7 GHs were shown to be upregulated in response to xylan compared to any of the other sugars, and of these, 5 GHs were additionally shown to be active upon growth on xylan by labeling with JJB384 (Supplementary Table 2 in Supplementary Data Sheet 1). In addition, EGIDFPOO_01993 (predicted glucan-branching enzyme) was only confirmed via ABPP.

### Novel Enzyme Activities of Carbohydrate-Active Enzymes Identified by Activity-Based Protein Profiling

#### Maltose-Forming α-Amylase and Deacetylase Activity of EGIDFPOO_00674

The putative GH57 family protein EGIDFPOO_00674 with uncertain function is potentially involved in xylan degradation as it was enriched with the probe JJB384 upon growth of cells on xylan and was likewise found to be upregulated in the proteome of cells grown on xylan compared to cells grown on D-glucose, Avicel^®^ cellulose or D-xylose. EGIDFPOO_00674 (70.6 kDa, 599 amino acids) contains a GH57 family domain spanning the amino acid residues 52–300, with E153 and D253 acting as catalytic residues, as predicted by HMMER analysis. This protein is conserved in many *Thermococcus* species (see Table 3) as revealed by sequence similarity search with BLASTP, but for most homologs no specific function is assigned, except for the maltose-forming amylase PY04_RS04545 from *Pyrococcus* sp. strain ST04 (Jung et al., 2014), which has a sequence identity of 69% to EGIDFPOO_00674. Several other amylases were identified via HHpred analysis as structural homologs. However, remote structural homologs with assigned functions as mannosidases, glucanotransferases or polysaccharide deacetylases/esterases were also identified (Supplementary Table 3 in Supplementary Data Sheet 1). Of note, the structurally similar regions of the homologs annotated as deacetylases roughly correspond to the GH57 domain region of EGIDFPOO_00674 with its catalytic residues.

**TABLE 3:**
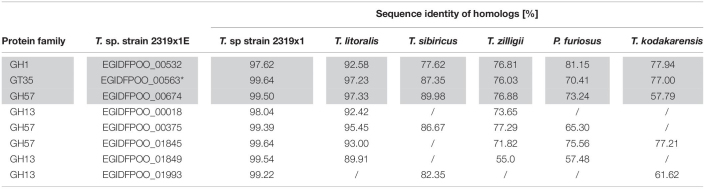
Distribution of glycosyl hydrolase homologs (according to BLASTP) of upregulated proteins identified by ABPP or comparative proteomics during growth on xylan in *Thermococcus* sp. strain 2319x1E in other members of the *Thermococcales*.

*Enzymes with proposed function in xylan hydrolysis are highlighted in gray.*

*Xylanolytic activity for the GT35 homolog was reported only for T. zilligii (Uhl and Daniel, 1999), however, as previously suggested (Rolland et al., 2002) and in accordance with our studies, the enzyme possesses glycogen phosphorylase activity (Supplementary Figure 7 in Supplementary Data Sheet 1).

**FIGURE 7:**
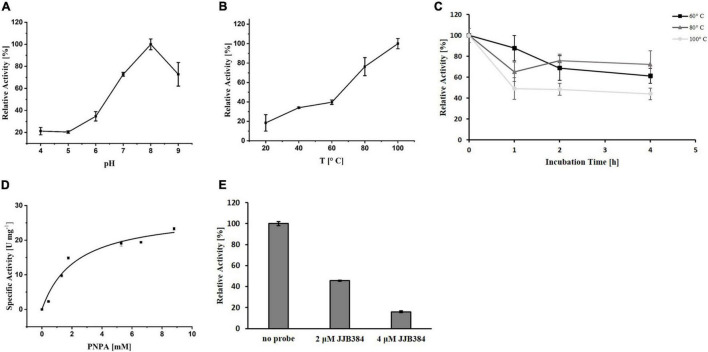
Characterization of heterologously expressed EGIDFPOO_00674 using PNPA as substrate. Effect of pH **(A)** and temperature **(B)** on the deacetylase activity of EGIDFPOO_00674. **(C)** Thermostability of EGIDFPOO_00674. The residual deacetylase activity of EGIDFPOO_00674 after preincubation at 60°C (black line, ■), 80°C (dark gray line, ▲) or 100°C (bright gray line, ●) for up to 4 h was determined. **(D)** Kinetic characterization of EGIDFPOO_00674 using PNPA (0–8.8 mM) as substrate (pH 8.0, 100 °C). **(E)** Inhibition of EGIDFPOO_00674 deacetylase activity by preincubation with JJB384 (2 and 4 μM). The kinetic characterization and the inhibition assays were performed at pH 8.0 and 100°C. Deacetylase activity was determined by measuring the increase in absorbance at 400 nm. All measurements were performed in triplicates.

To elucidate the precise function of EGIDFPOO_00674, the enzyme was heterologously expressed in *E. coli* and purified (Supplementary Figure 5 in Supplementary Data Sheet 1). The homolog PY04_RS04545 from *Pyrococcus* sp. strain ST04 has been demonstrated to efficiently cleave α-1,6-linked maltose residues from β-cyclodextrins and thus exhibits maltose-forming α-amylase activity (Jung et al., 2014). Indeed, purified EGIDFPOO_00674 showed comparable activity with 6-*O*-α-maltosyl-β-cyclodextrin as substrate (14.1 U mg^–1^ protein, data not shown). Interestingly, deacetylase activity was also observed by using the artificial substrate PNPA, indicating that this enzyme might function as a bifunctional α-amylase/carbohydrate deacetylase. The highest deacetylase activity was detected at a pH of 8.0 (Figure 7A) and a temperature of 100°C (Figure 7B). Remarkably, EGIDFPOO_00674 retained 44% (100°C) to 72% (80°C) of its deacetylase activity after 4 h of incubation at the respective temperature (Figure 7C). A V_*max*_ of 28.19 U mg^–1^ protein and a *K*_*m*_ of 2.57 mM was determined for the hydrolysis of PNPA by kinetic measurements (Figure 7D). Furthermore, the deacetylase activity of EGIDFPOO_00674 was efficiently decreased in a concentration-dependent manner to 46% (2 μM JJB384) and 16% (4 μM JJB384) residual activity by preincubation with the α-glucosidase probe, thus confirming the specific covalent binding of the ABP to the active site of the enzyme (Figure 7E).

#### Promiscuous β-Glycosidase Activity of EGIDFPOO_00532

EGIDFPOO_00532 (49.32 kDa, 420 amino acids) was predicted as β-glucosidase by Swiss-Prot annotation and is the only β-specific GH in *Thermococcus* sp. strain 2319x1E that was enriched with the probe JJB384. Indeed, a GH1 family domain spanning the amino acids 1–407 (e-value 1.2 × 10^–90^) was predicted for this enzyme by HMMER analysis. EGIDFPOO_00532 is conserved in many different species of the *Thermococcales* (see Table 3), as determined by BLASTP searches and respective homologs have proposed functions as β-glucosidases or β-galactosidases (Supplementary Figure 4 in Supplementary Data Sheet 1). The majority of close structural homologs are predicted by HHpred to possess either β-glucosidase or β-galactosidase activity, whereas β-xylanase activity was only reported for more remote (probability > 95%) structural homologs (Supplementary Table 4 in Supplementary Data Sheet 1). Although no secretion signals or transmembrane regions were predicted for EGIDFPOO_00532, a close homolog from *P. horikoshii* (80.91% sequence identity) has been characterized as a putatively membrane bound β-glycosidase capable of hydrolyzing different alkyl-β-glycosides (Akiba et al., 2004).

**FIGURE 8:**
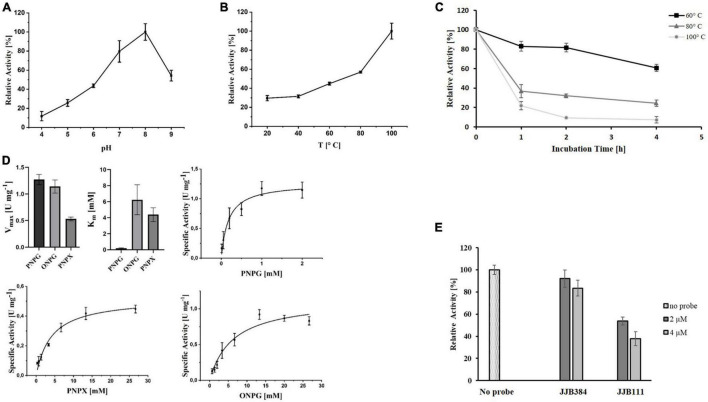
Characterization of heterologously expressed EGIDFPOO_00532. Effect of pH **(A)** and temperature **(B)** on the β-xylosidase activity of EGIDFPOO_00532. **(C)** Thermostability of EGIDFPOO_00532. The residual β-xylosidase activity of EGIDFPOO_00532 after preincubation at 60°C (black line, ■), 80°C (dark gray line, ▲) or 100°C (bright gray line, ●) for up to 4 h was determined. **(D)** Kinetics characterization of EGIDFPOO_00532 with the nitrophenyl substrates PNPG, ONPG and PNPX (pH 8.0, 100°C). **(E)** Inhibition of β-xylosidase activity of EGIDFPOO_00532 with 2 or 4 μM JJB384 and JJB111, respectively.

Since EGIDFPOO_00532 was classified as a GH1 family β-glucosidase, its hydrolytic activity was determined using the chromogenic substrate PNPG (β-glucosidase activity), alongside with the further substrates ONPG (β-galactosidase activity) and PNPX (β-xylosidase activity) after heterologous expression of the enzyme in *E. coli* and denaturing purification from inclusion bodies (Supplementary Figure 6 in Supplementary Data Sheet 1). The enzyme possesses broad substrate specificity and showed activity with all three substrates. We first determined the pH and temperature optimum monitoring the β-xylosidase activity of EGDIFPOO_00532. Highest activity with PNPX was observed at a pH of 8.0 (Figure 8A) and a temperature of 100°C (Figure 8B). The thermostability of EGDIFPOO_00532 was determined by preincubation of the enzyme up to 4 h at 60, 80, and 100°C, revealing 61, 32, and 7% residual enzyme activity, respectively (Figure 8C). The kinetic characterization was performed for the three substrates at 100°C and pH 8.0, revealing highest activity for PNPG with a V_*max*_ of 1.27 U mg^–1^ protein and a *K*_*m*_ of 0.18 mM. Slightly less activity was determined for ONPG (V_*max*_ = 1.14 U mg^–1^ protein, *K*_*m*_ = 6.23 mM) and roughly half of the activity for PNPX with a V_*max*_ of 0.53 U mg^–1^ protein and a *K*_*m*_ of 4.38 mM (Figure 8D). Accordingly, EGIDFPO_00532 is a promiscuous β-glucosidase with additional β-xylosidase and β-galactosidase activity. The β-xylosidase activity of EGDIFPOO_00532 was furthermore shown to be reduced by preincubation with the α- and β-selective glucosidase probes JJB384 and JJB111, respectively. While the application of 4 μM of the α-selective glucosidase probe JJB384 only resulted in a slight inhibition of the β-xylosidase activity (85% activity compared to the untreated control), a considerable decrease in activity to 38% was observed upon preincubation with 4 μM of the β-glucosidase probe JJB111 (Figure 8E). This is in agreement with the identification of EGDIFPOO_00532 using JJB384, but furthermore confirms nicely the α- and β-preference of both probes.

## Discussion

The archaeal metabolism resembles that of Bacteria and lower Eukaryotes in complexity, but Archaea are characterized by unique metabolic features as well as modifications in common metabolic pathways. Their metabolic potential in combination with their prevalence under extreme environmental conditions renders their enzyme repertoire of particular interest for industrial use. Despite years of research, the metabolic complexity of Archaea has yet to be resolved and deeper understanding of their metabolic pathways is only available for a few archaeal model organisms. In order to unravel the metabolic potential of Archaea and their extremozymes for biotechnological applications, alternative approaches can be helpful. ABPP is an attractive methodology in many different fields of research and its application enables to bridge the gap between “native activity measurements” which do not provide information about the proteins that contribute to this activity, and polyomic studies, which lack information about the function of the genes and the activity state of the proteins identified. Although well established for eukaryotic (Niphakis and Cravatt, 2014; Morimoto and van der Hoorn, 2016) and bacterial (Keller et al., 2020) organisms, this methodology has only recently been applied in the Archaea for the first time and turned out to be likewise suitable for the identification of serine hydrolases under the extreme conditions many of the so far cultivated Archaea thrive in (Zweerink et al., 2017).

We herein demonstrated the identification of enzymes involved in xylan degradation in the hyperthermophilic *Thermococcus* isolate strain 2319x1E by ABPP, thereby expanding the applicability of this methodology by the identification of glycoside hydrolases in Archaea. We first showed that the organism, which was isolated from the same enrichment as the previously described isolate strain 2319x1 by *in situ* cultivation techniques (Gavrilov et al., 2016), likewise uses xylan as well as crystalline cellulose as its sole carbon and energy source. The utilization of these carbon sources is rare among the Archaea and has so far only been reported for a few organisms. Archaeal growth in pure culture on crystalline cellulose for example has only been demonstrated for *Desulfurococcus fermentans* (Perevalova et al., 2005) and some haloarchaeal species (Sorokin et al., 2015). Reports of Archaea growing on xylan on the other hand are limited to crenarchaeal species, such as *Thermosphaera aggregans* (Huber et al., 1998), *Sulfolobus solfataricus* (Cannio et al., 2004) and *Acidilobus saccharovorans* (Prokofeva et al., 2009). These two *Thermococcus* strains hence are the first representatives of hyperthermophilic Euryarchaea growing with xylan as growth substrate. Accordingly, the induction of xylanolytic activity, which was measured predominantly in cell membrane fractions, was demonstrated for *Thermococcus* sp. strain 2319x1E. Following these basic analyses, we then decided for a combined (chemo)proteomics approach using a comparative label-free full proteome analysis to detect enzymes involved in xylan degradation as well as ABPP in order to confirm their activity upon growth on xylan. This enabled the identification of a subset of upregulated proteins with activity toward the employed ABP(s). Since JJB384 is glycosidase-selective with a preference for targeting retaining α-glucosidases, it is likely that the identified CAZymes are somehow involved in the hydrolysis of the complex xylan polymer. To confirm this assumption, we further characterized two of the candidates, i.e., EGIDFPOO_00532 (GH1) and EGIDFPOO_00674 (GH57).

Based on our findings, we suggest that EGIDFPOO_00532 (GH1) and EGIDFPOO_00674 (GH57) are involved in xylan degradation, thus contributing to the xylanolytic activity in *Thermococcus* sp. strain 2319x1E cells that was shown to be inducible upon growth on xylan, predominantly localized in cell membrane fractions (Figure 3) and targeted by the ABPs JJB384 (preferably targeting α-specific GH) and JJB111 (preferably targeting β-specific GH) (Figure 5). Both glycosidases which have been described in this work display high thermostability and retain considerable activity over several hours at 60°C (EGIDFPOO_00532, Figure 8C) or even 100°C (EGIDFPOO_00674, Figure 7C).

EGIDFPOO_00674 is predicted to belong to the comparatively scarcely described GH57 family, which is frequently found in the genomes of hyperthermophilic Archaea, often occurring jointly with GH13 family enzymes (Janecek et al., 1999). So far, α-amylase, α-galactosidase, amylopullulanase, branching enzyme and 4-α-glucanotransferase activities have been reported for characterized GH57 family members, and it is likely that additional functionalities within this family will be described in the future (Blesák and Janeček, 2012). We could demonstrate that this protein can efficiently cleave α-1,6-linked maltose residues from β-cyclodextrins like it was reported for the closely related homolog from *Pyrococcus* sp. strain ST04 (Jung et al., 2014). Furthermore, the decent hydrolysis of PNPA indicates a potential role as carbohydrate esterase/deacetlyase (Figure 7), which cleaves acetyl residues from the xylan backbone and makes it thus more accessible for xylanases and xylosidases, respectively. There are several examples for bifunctional enzymes with two different glycoside hydrolase functions (Shi et al., 2010; Ferrara et al., 2014; Liang et al., 2018). Especially for xylanases, bi- or multifunctionality is common, including acetyl ester-xyloside hydrolases (Khandeparker and Numan, 2008; Cao et al., 2019). However, as far as we know, a bifunctional enzyme with maltose-forming α-amylase and deacetylase activity has not been reported yet. In addition, we suggest that for EGIDFPOO_00674 both functionalities are located at the same site within the GH57 domain or at least in close vicinity to each other, since the deacetylase activity is significantly decreased upon binding of the α-glucosidase-selective ABP JJB384 to this enzyme (Figure 7).

For EGIDFPOO_00532 we elucidated promiscuous β-glucosidase activity with significant side activity on galactosides and xylosides (Figure 8) contributing to the breakdown of xylan in *Thermococcus* sp. strain 2319x1E. Although no secretion signals or transmembrane domains were predicted for EGIDFPOO_00532, it appears plausible that EGIDFPOO_00532 is indeed a membrane bound protein, since a close homolog in *P. horikoshii* is described as membrane associated (Matsui et al., 2000; Akiba et al., 2004) and its heterologous expression led to inclusion body formation. Thus, it is potentially exposed to the cell surface and can access large xylan polymers and xylooligosaccharides. While it is common for glycosidases of the GH1 family to possess both β-glucosidase and β-galactosidase activity, β-xylosidase/xylanase activity is unusual for this family (He and Withers, 1997). However, there are examples of other GH1 family hydrolases, which likewise show decent activity with the xylosidase substrate PNPX (Liu et al., 2018; Yin et al., 2020) and promiscuous glycoside hydrolases of other GH families, mainly from the GH family 3, that show β-glucosidase as well as β-xylosidase activity, have also been reported (Zhou et al., 2012; Gruninger et al., 2014; Patel et al., 2018).

Analysis of the prevalence of homologs of the CAZymes discussed in this work (EGIDFPOO_00532 and EGIDFPOO_00674) across other members of the *Thermococcales*, such as *T. kodakarensis*, *T. litoralis*, or *T. sibiricus*, revealed that these enzymes are widely distributed (Table 3), suggesting that these strains might be capable of utilizing xylan as carbon source. Furthermore, it remains to be investigated whether and how the other candidates that were identified via ABPP and/or comparative proteomics contribute to the xylanolytic activity of *Thermococcus* sp. strain 2319x1E. For instance, the enzymes EGIDFPOO_00018, EGIDFPOO_00375, EGIDFPOO_00563, EGIDFPOO_01845, EGIDFPOO_01849, and EGIDFPOO_01993 with predicted α-specific functionalities could possibly be involved in the cleavage of (α-linked) side branches of different xylans, such as mannose, arabinofuranose or glucuronic acid. Alternatively, we may have identified these candidates due to the co-occurrence of xylans with other polymers in nature, such as other hemicelluloses, starch or pectin (Tharanathan et al., 1987; Rodrigues Mota et al., 2018). Consistent with this assumption we expressed and purified EGIDFPOO_00563 and demonstrated glycogen phosphorylase activity, i.e., the formation of glucose 1-phosphate from glycogen, maltodextrin and starch in presence of phosphate, but no glycosidase activity was observed with xylan as substrate (Supplementary Figure 7 in Supplementary Data Sheet 1). As mentioned above, this enzyme belongs to the GT 35 family (EC 2.4.1.1) and the homolog in *T. zilligii* (76% sequence identity) was erroneously reported to possess endo-xylanase activity (Uhl and Daniel, 1999; Rolland et al., 2002).

In general, it should be kept in mind that the outcome of such ABPP studies strongly depends on the probes used. The ABPs employed in this study have been reported to preferably target exo-glycosidases (Kallemeijn et al., 2012; Jiang et al., 2016; Husaini et al., 2018) with preference for α-linked (JJB384) or β-linked (JJB111) substrates, which is thus in line with the findings of this study. However, xylans display a complex structure composed of a diverse set of monosaccharides connected by many different types of glycosidic bonds and thus, a broad and heterogenous set of hydrolytic enzymes is required for its degradation (Figure 1). However, we herein clearly demonstrated the benefits of (chemo)proteomics approaches to identify key players involved in polysaccharide degradation. We successfully showed that ABPP with glycoside hydrolase-specific ABPs can be utilized for the functional characterization of CAZymes in (hyper)thermophilic Archaea and thus further extended the scope of ABPP in extremophiles. ABPP therefore represents a promising novel approach for the identification of extremozymes, which may also be of biotechnological interest.

## Conclusion

We successfully established ABPP with glycoside hydrolase-specific ABPs for the identification of active CAZymes in hyperthermophilic Archaea, here the novel isolate *Thermococcus* sp. strain 2319x1E. The challenging task of identifying novel biocatalysts from (hot) environments has frequently been addressed using (functional) metagenomics (Voget et al., 2003; Ferrer et al., 2016). These approaches, however, lack the option of verifying enzyme activities under physiological conditions. In addition, mesophilic bacteria that possess fundamental differences, for example, in information processing such as transcription (e.g., different eukaryal-like promoter structure and RNA polymerase), translation, post-translational modification (e.g., glycosylation) and protein transport (e.g., signal peptides) are typically used for functional screening. Therefore, this selective approach does not cover the entire biological diversity.

Due to its high abundancy, xylan, for example, is an attractive and cheap resource for various industrial applications in the food, pulp and paper industry and in the production of renewable fuels and chemicals (Butt et al., 2007; Goluguri et al., 2012; Menon and Datta, 2017; Walia et al., 2017), which comes along with ecological and economic benefits (Ayyachamy and Vatsala, 2007; Woldesenbet et al., 2012; Chandel(ed.), 2013; Motta et al., 2013). For biomass conversion, biocatalysts from thermophiles and hyperthermophiles are of particular interest since they generally possess high thermostability, often in combination with high general robustness and stability under harsh reaction conditions, including the presence of detergents and organic solvents (Egorova and Antranikian, 2005; Elleuche et al., 2014; Schocke et al., 2019). Moreover, it is noteworthy that industrial processes at elevated temperatures are often desired due to higher substrate solubility and accessibility as well as a lower risk of contamination (Cokgor et al., 2009). Therefore, the application of alternative screening strategies, such as ABPP in extremophilic environments, represents an alternative approach to address biological diversity and to screen for novel biocatalysts.

## Data Availability Statement

The datasets presented in this study can be found in online repositories. The names of the repository/repositories and accession number(s) can be found below: https://www.ebi.ac.uk/pride/archive/, PXD026056 (Vizcaíno et al., 2016); https://www.ncbi.nlm.nih.gov/, LR778300.

## Author Contributions

IK, AE, and KZ performed the strain isolation, initial cultivation, and genome analysis. TK performed cultivation of the cells, determination of native enzyme activity, cloning and heterologous expression, purification as well as biochemical characterization of the genes of interest. SN and TK performed *in vivo* chemical labeling, protein extraction and affinity enrichment experiments. SN performed the MS analysis experiments. AA, TB, DW, and JK performed the genome sequencing, assembly and annotation. JJ synthesized ABPs in the HO laboratory. CB, FK, IK, HO, BS, and MK supervised the study. TK and SN wrote the manuscript under supervision of IK, MK, and BS. All authors contributed to the article and approved the submitted version.

## Conflict of Interest

The authors declare that the research was conducted in the absence of any commercial or financial relationships that could be construed as a potential conflict of interest.

## Publisher’s Note

All claims expressed in this article are solely those of the authors and do not necessarily represent those of their affiliated organizations, or those of the publisher, the editors and the reviewers. Any product that may be evaluated in this article, or claim that may be made by its manufacturer, is not guaranteed or endorsed by the publisher.

## Footnotes

1
http://www.cazy.org/

## Abbreviations

ABP: activity-based probe
ABPP: activity-based protein profiling
CAZymes: carbohydrate-active enzymes
CMC: carboxymethyl cellulose
DAPI: 4′,6-diamidino-2-phenylindole
DNSA: 3,5-dinitrosalicylic acid
HRP: horseradish peroxidase
IPTG: isopropyl- β -D-thiogalactopyranosid
LB: lysogeny broth
LFQ: label-free quantification
M β CD: 6-*O*- α -maltosyl- β -cyclodextrin
MS: mass spectrometry
ONPG: *ortho*-nitrophenyl- β -D-galactopyranoside
PBS: phosphate buffered saline
PNP: *para*-nitrophenol
PNPA: *para*-nitrophenyl-acetate
PNPG: *para*-nitrophenyl- β -D-glucopyranoside
PNPX: *para*-nitrophenyl- β -D-xylopyranoside.
