# Supplementary material for: Activity-Based Protein Profiling for the Identification of Novel Carbohydrate-Active Enzymes Involved in Xylan Degradation in the Hyperthermophilic Euryarchaeon Thermococcus sp. Strain 2319x1E
Source: Front Microbiol. 2022 Jan 12;12:734039. doi: 10.3389/fmicb.2021.734039 (PMC8790579; doi:10.3389/fmicb.2021.734039)
Supplement: Supplementary file 1 [file Data_Sheet_1.pdf]

Activity-based protein profiling for the identification of novel carbohydrate-active enzymes involved in xylan degradation in the hyperthermophilic Euryarchaeon *Thermococcus* sp. strain 2319x1E

Thomas Klaus[1#], Sabrina Ninck[2#], Andreas Albersmeier[3] Tobias Busche[3], Daniel Wibberg[3], Jianbing Jiang[4§], Alexander G. Elcheninov[5], Kseniya S. Zayulina[5], Farnusch Kaschani[2], Christopher Bräsen[1], Herman S. Overkleeft[4], Jörn Kalinowski[3], Ilya V. Kublanov[5], Markus Kaiser[2], Bettina Siebers[1]

[1]Molecular Enzyme Technology and Biochemistry (MEB), Environmental Microbiology and Biotechnology (EMB), Faculty of Chemistry, Centre for Water and Environmental Research (CWE), University of Duisburg-Essen, Essen, Germany

[2]Chemical Biology, Center of Medical Biotechnology, Faculty of Biology, University of Duisburg-Essen, Germany

[3]Center for Biotechnology (CeBiTec), Bielefeld University, Bielefeld, Germany

[4]Bio-organic synthesis, Leiden Institute of Chemistry, University of Leiden, Leiden, Netherlands

[5]Winogradsky Institute of Microbiology, Research Center of Biotechnology, Russian Academy of Sciences, Moscow, Russia

Corresponding authors: Bettina Siebers and Markus Kaiser

#both authors contributed equally

§current address: Health Science Center, School of Pharmacy, Shenzhen University, Shenzhen, China

1 Supplementary Figures

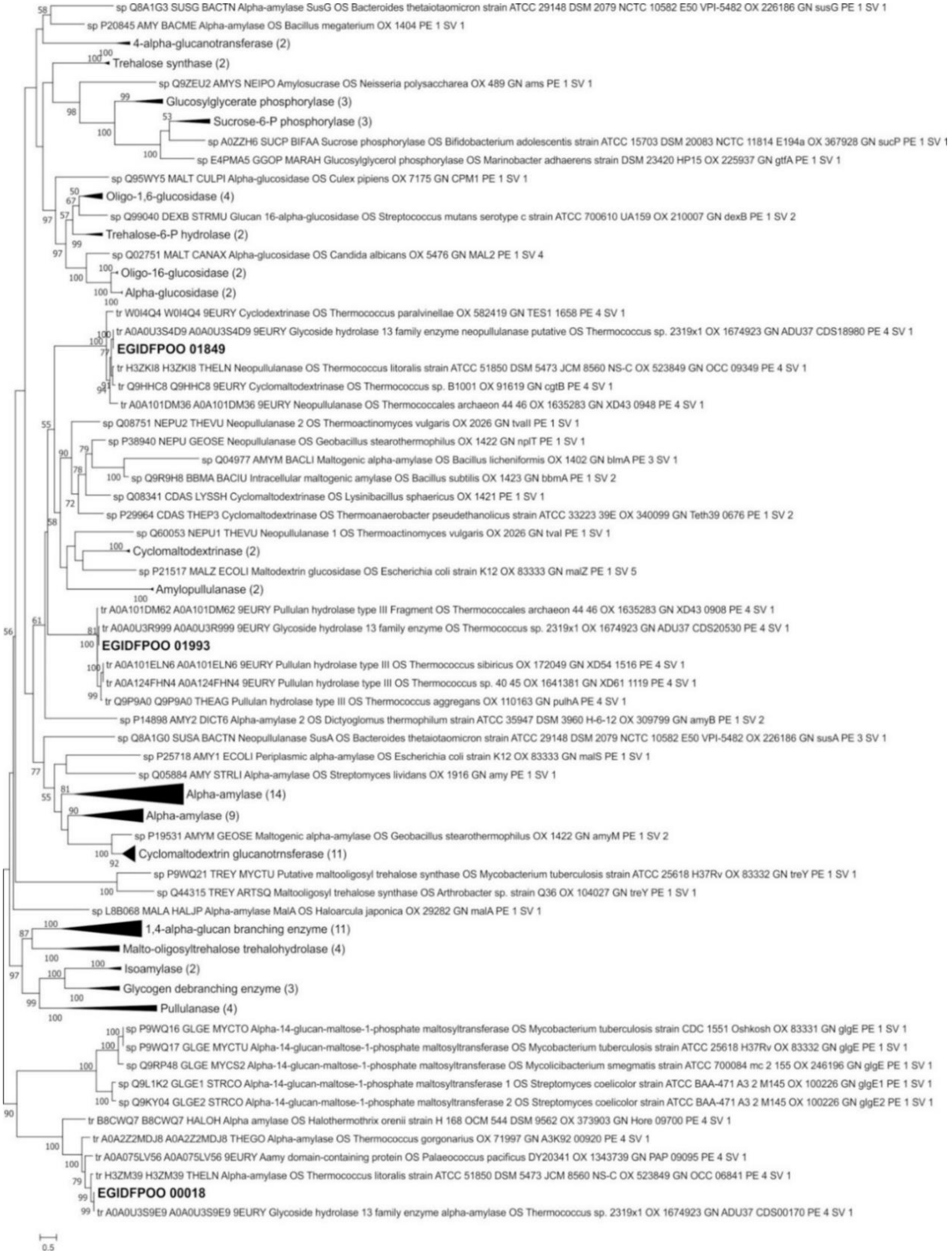

Supplementary Figure 1 ‖ Phylogenetic position of the GH13 family enzymes EGIDFPOO_00018, EGIDFPOO_01849 and EGIDFPOO_01993 from *Thermococcus* sp. strain 2319x1E, top 5 BLAST homologs and all proteins from the Swiss-Prot database (evidence at protein level only) affiliated to the GH13 family.

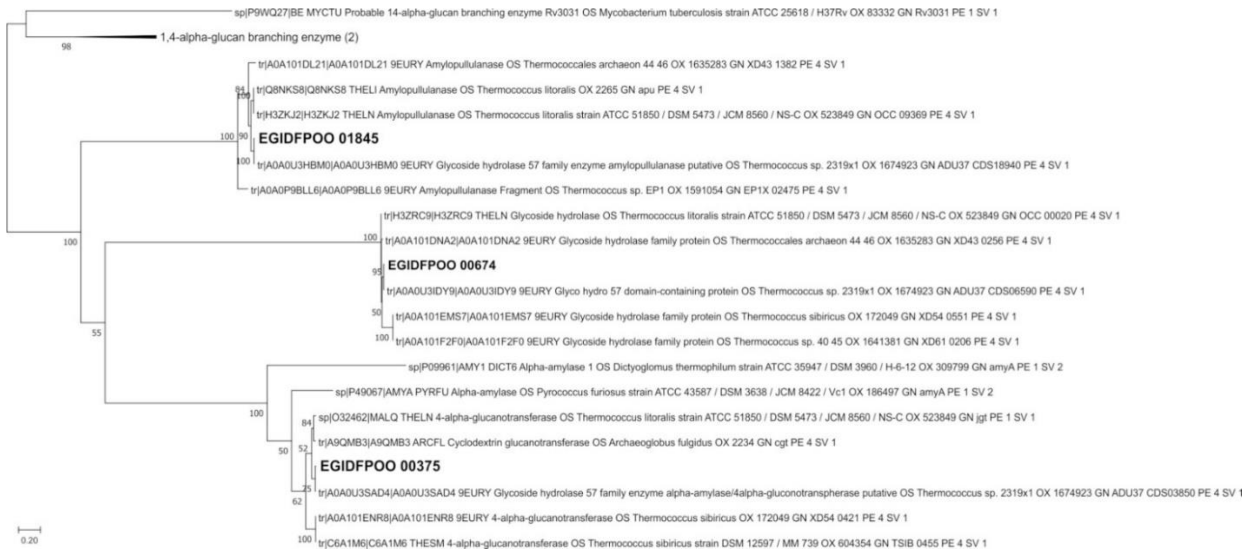

Supplementary Figure 2 ‖ Phylogenetic position of the GH57 family enzymes EGIDFPOO_00375, EGIDFPOO_00674 and EGIDFPOO_01845 from *Thermococcus* sp. strain 2319x1E, top 5 BLAST homologs and all proteins from the Swiss-Prot database (evidence at protein level only) affiliated to the GH57 family.

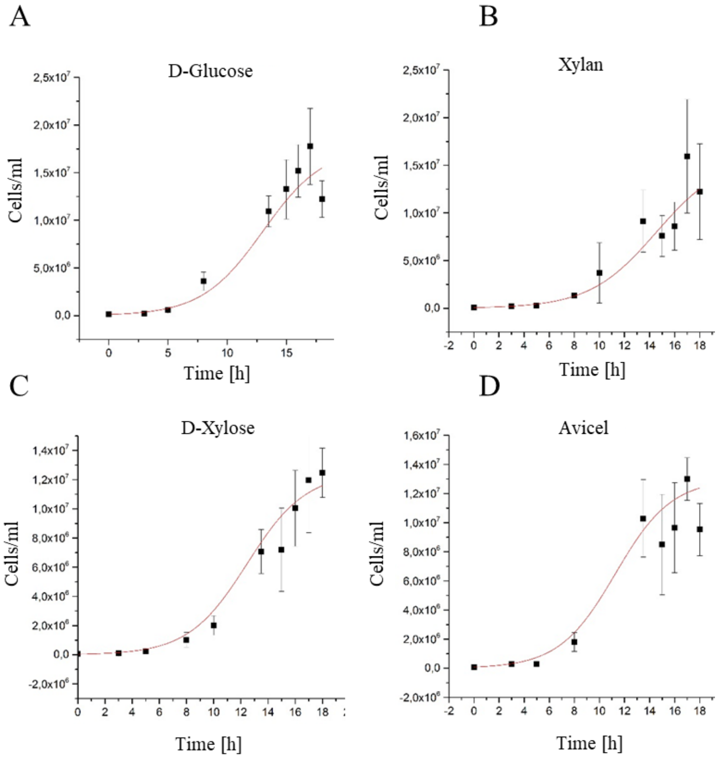

Supplementary Figure 3 ‖ Growth of *Thermococcus* sp. strain 2319x1E on D-glucose (A), xylan (B), D-xylose (C) or Avicel (D) as carbon source. Modified pfennig medium at pH 7.0 with 0.1 g l^{-1} yeast extract and 1 g l^{-1} of the respective sugar was used for growth. Cells were stained with DAPI, counted on a Zeiss Axioscope and the resulting cell numbers were fitted in Origin 2019 (OriginLab corporation, USA) using the sigmoidal fit function sfit1.

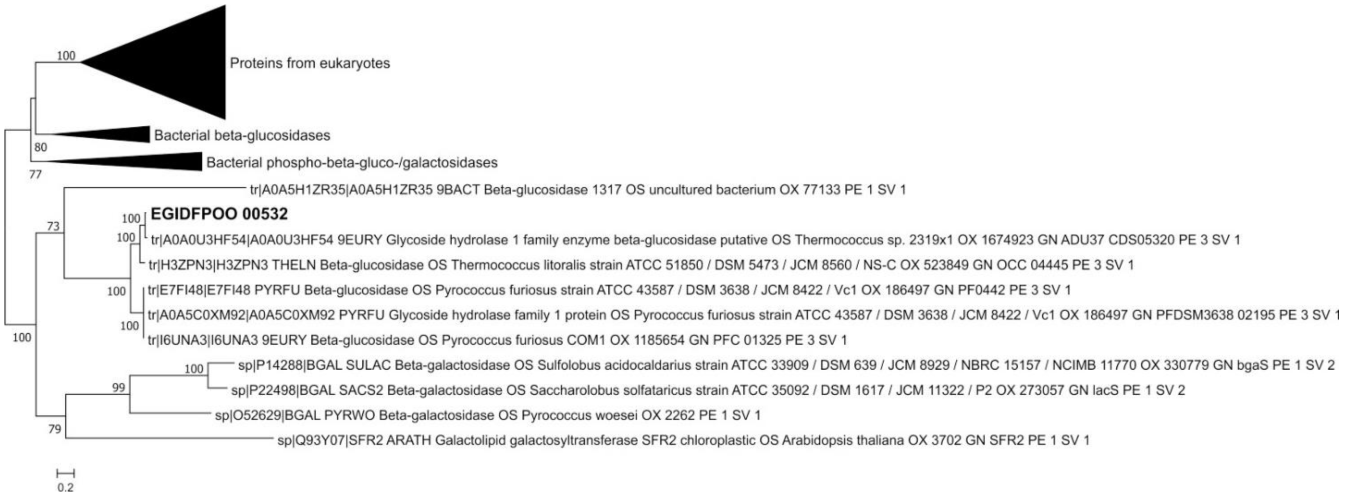

Supplementary Figure 4 ‖ Phylogenetic position of the GH1 family enzyme EGIDFPOO_00532 from *Thermococcus* sp. strain 2319x1E, top 5 BLAST homologs and all proteins from the Swiss-Prot database (evidence at protein level only) affiliated to the GH1 family.

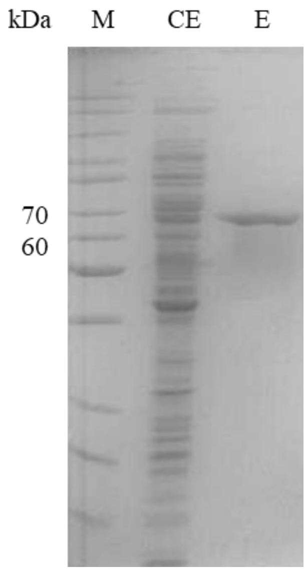

Supplementary Figure 5 ‖ Purification of EGIDFPOO_00674 after heterologous expression in *E. coli*. The recombinant protein was purified from the soluble crude cell extract (CE) by immobilized metal affinity chromatography (IMAC). A protein band corresponding to a size of ~70 kDa is present in the elution fraction (E). (M) Marker, PageRuler^{TM} Unstained Protein Ladder.

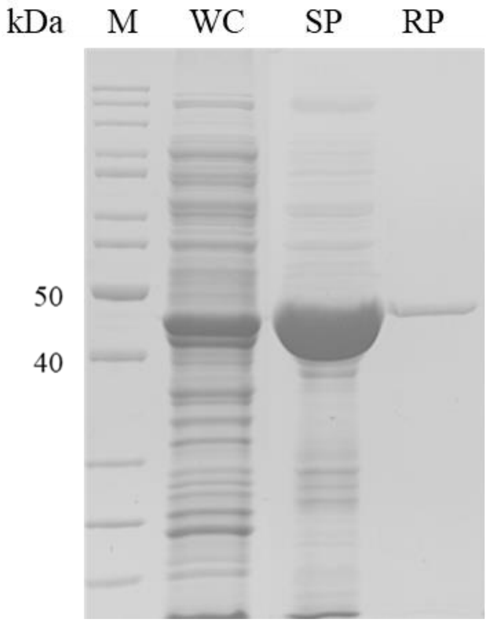

Supplementary Figure 6 ‖ Purification of EGIDFPOO_00532 from inclusion bodies after heterologous expression in *E. coli*. The protein was solubilized from the pellet fraction obtained after sonication with a buffer containing 2 M urea at pH 12.5. The solubilized proteins (SP) were refolded by dilution into a pH 8.0 buffer containing 10% (w/v) sucrose and 2 M urea and were subjected to a heat precipitation at 80° C for 20 min. The refolded protein (RP) has a size of about ~45 kDa. (WC): whole cell extract; (M), Marker PageRuler^{TM} Unstained Protein Ladder.

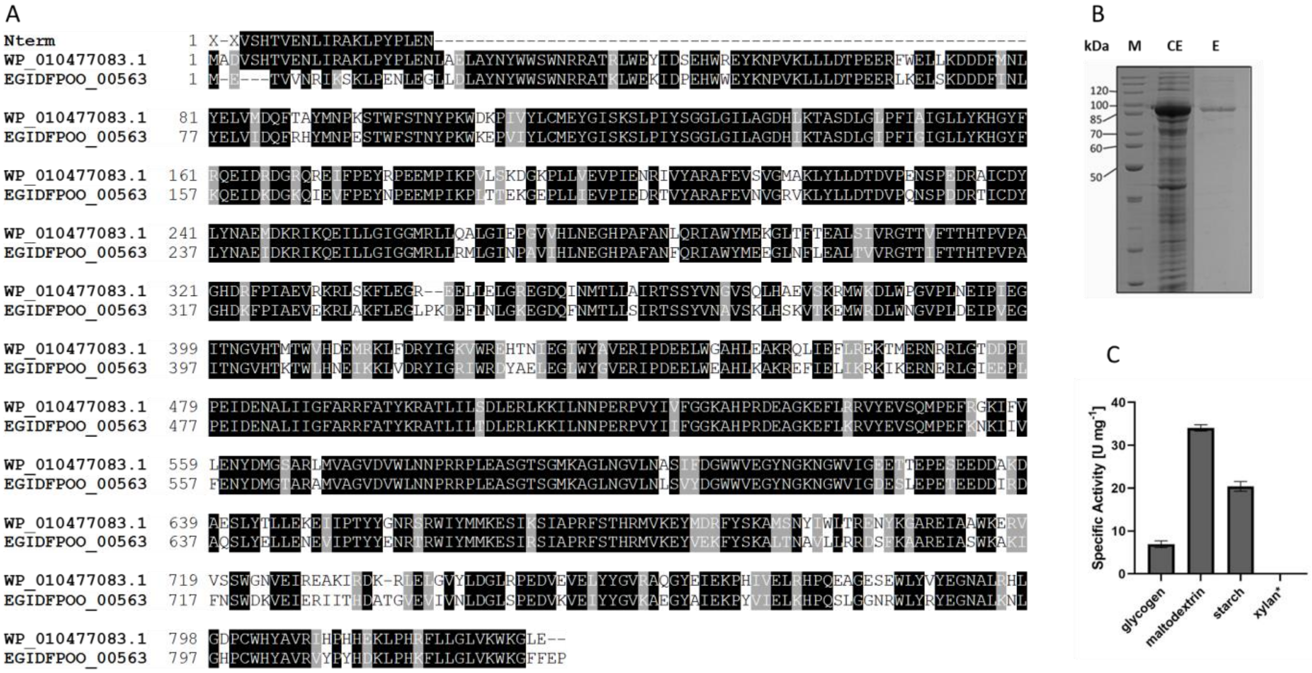

Supplementary Figure 7 ‖ Analysis of the GT35 family maltodextrin phosphorylase EGIDFPOO_00563. **(A)** Multiple Sequence Alignment generated with the N-terminal protein Sequence of the first putative archaeal xylanase from *Thermococcus zilligii* (Uhl *et al.,* 1999), the most suitable BLAST hit from the NCBI database (https://blast.ncbi.nlm.nih.gov/Blast.cgi, WP_010477083.1) and the proteinsequence of EGIDFPOO_00563. The alignment was constructed with T-Coffee (http://tcoffee.crg.cat/apps/tcoffee/do:regular) and Boxshade (http://www.ch.embnet.org/software/BOX_form.html). Identical corresponding amino acids are marked with a black box, whereas similar amino acids are marked with a grey box. **(B)** Purification of EGIDFPOO_00563 after heterologous expression in *E. coli*. The recombinant protein was purified from the soluble crude cell extract (CE) by heat precipitation (80° C, 20 min) and immobilized metal affinity chromatography (IMAC). A protein band corresponding to a size of ~100 kDa is present in the elution fraction (E). (M) Marker, PageRuler™ Unstained Protein Ladder. **(C)** Specific activities of EGIDFPOO_00563 for the substrates glycogen, maltodextrin, starch and xylan. The phosphorylase activity with glycogen, maltodextrin and starch was determined by quantification of glucose 1-phosphate using phosphoglucomutase and glucose-6-phosphate dehydrogenase as auxiliary enzymes. Endo-xylanase activity (xylan*) was determined using the DNSA assay, however, no formation of reducing sugars was observed.

2 Supplementary Tables

Supplementary Table 1 ‖ Comparison of the CAZymes present in the genomes of *Thermococcus* sp strain 2319x1 and strain 2319x1E.

CAZy domain	Function Swissprot	strain_2319x1	strain 2319x1E	Identity
GT55		lcl|CP012200.1_prot_ALV61890.1_191	EGIDFPOO_00184	100
		lcl|CP012200.1_prot_ALV63315.1_1616	EGIDFPOO_01570	99.808
GT35	Maltodextrin phosphorylase	lcl|CP012200.1_prot_ALV62263.1_564	EGIDFPOO_00563	99.639
GH57	amylopullulanase	lcl|CP012200.1_prot_ALV63593.1_1894	EGIDFPOO_01845	99.637
		lcl|CP012200.1_prot_ALV62017.1_318	EGIDFPOO_00304	99.553
GH13	neopullulanase	lcl|CP012200.1_prot_ALV63597.1_1898	EGIDFPOO_01849	99.542
CE1	putative carbohydrate esterase	lcl|CP012200.1_prot_ALV63041.1_1342	EGIDFPOO_01302	99.517
GH57		lcl|CP012200.1_prot_ALV62358.1_659	EGIDFPOO_00674	99.499
GT4		lcl|CP012200.1_prot_ALV63230.1_1531	EGIDFPOO_01440	99.43
GT39		lcl|CP012200.1_prot_ALV62042.1_343	EGIDFPOO_00332	99.417
GH57	alpha-amylase/ alpha-gluconotranspherase	lcl|CP012200.1_prot_ALV62084.1_385	EGIDFPOO_00375	99.393
GH57	1,4alpha-branching enzyme	lcl|CP012200.1_prot_ALV62996.1_1297	EGIDFPOO_01266	99.388
GH130	beta-1,4 mannooligosaccharide phosphorilase	lcl|CP012200.1_prot_ALV61989.1_290	EGIDFPOO_00274	99.286
GH13		lcl|CP012200.1_prot_ALV63752.1_2053	EGIDFPOO_01993	99.219
CE10		lcl|CP012200.1_prot_ALV62685.1_986	EGIDFPOO_00955	99.211
GT66		lcl|CP012200.1_prot_ALV61904.1_205	EGIDFPOO_00197	99.016
GH57		lcl|CP012200.1_prot_ALV62233.1_534	EGIDFPOO_00534	98.626
GT2		lcl|CP012200.1_prot_ALV62109.1_410	EGIDFPOO_00399	98.611
GT5		lcl|CP012200.1_prot_ALV63599.1_1900	EGIDFPOO_01851	98.462
GT2		lcl|CP012200.1_prot_ALV61710.1_11	EGIDFPOO_00012	98.295
GH13	alpha-amylase	lcl|CP012200.1_prot_ALV61716.1_17	EGIDFPOO_00018	98.044
GT2		lcl|CP012200.1_prot_ALV63648.1_1949	EGIDFPOO_01900	98.006
CE10		lcl|CP012200.1_prot_ALV62860.1_1161	EGIDFPOO_01219	97.953
GT4		lcl|CP012200.1_prot_ALV62898.1_1199	EGIDFPOO_01181	97.878
GT81		lcl|CP012200.1_prot_ALV63409.1_1710	EGIDFPOO_01674	97.817
GH1	putative beta-glucosidase	lcl|CP012200.1_prot_ALV62231.1_532	EGIDFPOO_00532	97.619
GH122	alpha-glucosidase	lcl|CP012200.1_prot_ALV62474.1_775	EGIDFPOO_00753	97.586
GT2		lcl|CP012200.1_prot_ALV62310.1_611	EGIDFPOO_00612	97.5
GT2		lcl|CP012200.1_prot_ALV63858.1_2159	EGIDFPOO_02198	95.076
GT4		lcl|CP012200.1_prot_ALV63856.1_2157	EGIDFPOO_02196	93.333
GT66		lcl|CP012200.1_prot_ALV62046.1_347	EGIDFPOO_00336	90.369
		lcl|CP012200.1_prot_ALV63429.1_1730	EGIDFPOO_01702	72.784
GT4		lcl|CP012200.1_prot_ALV63888.1_2189	EGIDFPOO_02119	71.525
GT66		lcl|CP012200.1_prot_ALV63874.1_2175	EGIDFPOO_02109	37.951
GT2		cl|CP012200.1_prot_ALV63703.1_2004		
	putative glycogen debranching enzyme	lcl|CP012200.1_prot_ALV61988.1_289		
GH35	exo-beta D-glucosaminidase	lcl|CP012200.1_prot_ALV62426.1_727		
GH1	beta-galactosidase	lcl|CP012200.1_prot_ALV62433.1_734		
GH1	beta-galactosidase	lcl|CP012200.1_prot_ALV62443.1_744		
CE14		lcl|CP012200.1_prot_ALV62446.1_747		
GH13		lcl|CP012200.1_prot_ALV63800.1_2101		
GT2		lcl|CP012200.1_prot_ALV63855.1_2156		
GT4		lcl|CP012200.1_prot_ALV63859.1_2160		
GT4		lcl|CP012200.1_prot_ALV63871.1_2172		
GT2		lcl|CP012200.1_prot_ALV63872.1_2173		
GT4		lcl|CP012200.1_prot_ALV63873.1_2174		
GT2		lcl|CP012200.1_prot_ALV63886.1_2187		
		lcl|CP012200.1_prot_ALV63887.1_2188		
GH5+GH12+H12+CBM2	endoglucanase/endoxylanase	lcl|CP012200.1_prot_ALV63957.1_2258		
GT39		lcl|CP012200.1_prot_ALV63958.1_2259		

CAZy domain	Function Swissprot	strain_2319x1	strain 2319x1E	Identity
	amylo- α -1,6-glucosidase		EGIDFPOO_01323	
GH1	beta-glucosidase		EGIDFPOO_01324	
GT2			EGIDFPOO_02112	
GT4			EGIDFPOO_02121	
GT4			EGIDFPOO_02122	
GT4			EGIDFPOO_02135	
GT4			EGIDFPOO_02136	
GT4			EGIDFPOO_02137	
GT2			EGIDFPOO_02138	
GT2			EGIDFPOO_02139	
GT55			EGIDFPOO_02209	

Supplementary Table 2 ‖ Glycoside hydrolases identified by ABPP and/or comparative proteomics in *Thermococcus* sp. strain 2319x1E with their respective family affiliation, predicted function and reported substrates of characterized homologs. The sequence identity (SI) of the respective homologs to the *Thermococcus* sp. strain 2319x1E proteins is indicated. The proteins which are marked with a grey background have been characterized in this study, revealing promiscuous β-glucosidase activity with PNPG, PNPX and ONPG (EGIDFPOO_00532), maltose-forming α-amylase and deacetylase activity with PNPA (EGIDFPOO_00674) or glycogen phosphorylase activity with glycogen, maltodextrin or starch (EGIDFPOO_00563).

Gene	Identified by		Family	Predicted function (PFAM)	Characterized homolog	Reported substrates
	Comparative proteomics	ABPP				
EGIDFPOO_00018	+	-	GH13	α-amylase		
EGIDFPOO_00375	+	+	GH57	α-amylase/ α-gluconotranspherase	gtpK, *T. kodakarensis*, 78% SI (Ahmad *et al.*, 2014)	Maltooligosaccharides, starch
EGIDFPOO_00532	+	+	GH1	β-glucosidase	BGPh, *T. horikoshii*, 81% SI	β-D-saccharides, β-D-glucosides
EGIDFPOO_00563	+	+	GT35	Glycogen phosphorylase	*T. zilligii* strain AN1, 76% SI (Uhl & Daniel, 1999)*	Larch/oat spelt/birch wood xylan, wheat arabinoxylan,
EGIDFPOO_00674	+	+	GH57	GH family 57 protein	Py04_0872, *P.* sp ST04, 69 % SI (Jung *et al.*, 2014)	6-O-α-maltosyl-β-cyclodextrin,
EGIDFPOO_00753	(+)**	+	GH122	alpha-glucosidase		
EGIDFPOO_01845	+	+	GH57	GH family 57 protein, putative amylopullulanase	Tk1770, *T. kodakarensis*, 55% SI (Han et al. 2013)	
EGIDFPOO_01849	+	-	GH13	GH13 family protein, putative neopullulanase		Pullulan, α-cyclodextrine
EGIDFPOO_01993	-	+	GH13	1,4-α-glucan branching enzyme GlgB	TK_RS04810, *T. kodakarensis*, 62% SI (Sun et al. 2015)	Soluble starch, pullulan

* In accordance with previous reports *(Rolland et al., 2002)* and our studies, the xylanase activity for the *T. zilligii* homolog (Uhl & Daniel, 1999) has erroneously been reported.
**EGIDFPOO_00753 was slightly upregulated in xylan grown cells compared to D-glucose and Avicel® cellulose, but slightly downregulated compared to D-xylose.

Supplementary Table 3 ‖ Structural homologs of EGIDFPOO_00674 predicted with HHpred (Zimmermann et al. 2018). Remote homologs with deacetylase activity are highlighted in grey.

Hit	Function	Origin	Probability [%]	E-value	Score
4CMR_A	glycosyl hydrolase/deacetylase family protein; GH 57<br>exo-type maltose-forming amylase	*Pyrococcus sp. strain* ST04	100	2.8e-119	1016.49
3N98_A	α-amylase, GH57 family; GH57 family member, branching enzyme, transferase	*Thermococcus kodakarensis*	100	2.4e-31	300.44
5WU7_A	amylase, glycogen branching enzyme	*Pyrococcus horikoshii*	100	3.7e-32	307.05
2B5D_X	α-Amylase; (beta/alpha)7 barrel,	*Thermotoga maritima*	99.97	1.4e-28	275.94
3P0B_A	Glycoside Hydrolase GH57, glycogen branching, transferase	*Thermus thermophilus*	99.97	1.6e-28	276.2
1K1X_B	4-α-glucanotransferase	*Thermococcus litoralis*	99.95	2e-25	256.64
5JM0_A	α-mannosidase	*Saccharomyces cerevisiae* S288C	99.26	2.2e-9	131.46
6B9O_A	α-mannosidase	*Canavalia ensiformis*	99.21	2.2e-9	129.14
2WYH_B	family GH38 α-mannosidase	*Streptococcus pyogenes*	99.08	4.5e-9	126.02
3RXZ_D	Polysaccharide deacetylase	*Mycobacterium smegmatis*	98.68	0.000003	85.65
3S6O_C	Polysaccharide deacetylase family protein	*Burkholderia pseudomallei*	98.51	0.000021	81.24
4LY4_A	peptidoglycan deacetylase	*Helicobacter pylori*	98.37	0.000055	78.65
2CC0_B	Acetyl-xylan esterase	*Streptomyces lividans*	97.88	0.0012	61.56
4M1B_A	Polysaccharide deacetylase; carbohydrate esterase	*Bacillus anthracis*	97.85	0.0018	63.85

Supplementary Table 4 ‖ Structural homologs of EGIDFPOO_00532 predicted with HHpred (Zimmermann et al. 2018) . Remote homologs with xylanase activity are highlighted in grey.

**Hit**	**Function**	**Origin**	**Probability [%]**	**E-value**	**Score**
1VFF_A	β-glucosidase	*Pyrococcus horikoshii*	100	1.1e-47	345.5
4HA4_A	β-galactosidase	*Acidilobus saccharovorans*	100	1e-43	324.26
3WDP_Q	β-glucosidase	*Pyrococcus furiosus*	100	2.2e-43	321.04
1QVB_B	β-glycosidase	*Thermosphaera aggregans*	100	3.3e-43	320.2
2J78_B	β-glucosidase	*Thermotoga maritima*	100	3.8e-43	320.06
5YIF_A	β-galactosidase	*Bacillus sp.*	100	7e-43	318.93
4RE2_A	β-mannosidase	*Oryza sativa Indica*	100	1e-42	319.49
3F5L_B	β-glucosidase	*Oryza sativa Japonica*	100	1e-42	318.17
1PBG_A	β-galactosidase	*Lactococcus lactis*	100	3.2e-42	314.09
6KDC_A	β-glucosidase/galactosidase	*Fervidobacterium pennivorans*	100	2.6e-41	307.38
5OKA_B	β-galactosidase	*Geobacillus stearothermophilus*	100	6.3e-41	306.98
4PMU_B	Endo-1,4-β-xylanase	*Xanthomonas axonopodis pv. citri*	99.94	4.4e-25	194.16
4PMD_A	Endo-1,4-β-xylanase	*Caldicellulosiruptor bescii*	99.94	2.8e-25	193.73
5XZO_A	β-xylanase	*Bispora sp. strain* MEY-1	99.94	5.8e-25	191.6
1UR1_A	β-xylanase	*Cellvibrio mixtus*	99.94	6.5e-25	194.79
3EMZ_A	Endo-1,4-β-xylanase	*Bacillus sp. strain* BP-23	99.94	6.7e-25	190.82
1R85_A	Endo-1,4-β-xylanase	*Geobacillus stearothermophilus*	99.94	1.2e-24	193.06
3NIY_A	Endo-1,4-β-xylanase	*Thermotoga petrophila* RKU-1	99.94	1.3e-24	189.97
