# Supplementary material for: Activity-Based Protein Profiling for the Identification of Novel Carbohydrate-Active Enzymes Involved in Xylan Degradation in the Hyperthermophilic Euryarchaeon Thermococcus sp. Strain 2319x1E
Source: Front Microbiol. 2022 Jan 12;12:734039. doi: 10.3389/fmicb.2021.734039 (PMC8790579; doi:10.3389/fmicb.2021.734039)
Supplement: Supplementary file 2 [file Data_Sheet_2.PDF]

Overview

ACE project	Title
ACE_0391	Investigation of xylan degradation in *Thermococcus* sp 2319x1E
ACE_0491	Target-ID of JJB384 in *Thermococcus* sp. 2319x1E grown on xylan or xylose

ACE_0391

File legend

ACE ID	Organism	Organ/ cell line	Treatment/ experimental setup
ACE_0391_SN01	*Thermococcus* sp. 2319x1E	Whole organism	*Thermococcus* sp. 2319x1E grown on glucose_ replicate 1
ACE_0391_SN02	*Thermococcus* sp. 2319x1E	Whole organism	*Thermococcus* sp. 2319x1E grown on glucose_ replicate 2
ACE_0391_SN03	*Thermococcus* sp. 2319x1E	Whole organism	*Thermococcus* sp. 2319x1E grown on glucose_ replicate 3
ACE_0391_SN04	*Thermococcus* sp. 2319x1E	Whole organism	*Thermococcus* sp. 2319x1E grown on glucose_ replicate 4
ACE_0391_SN05	*Thermococcus* sp. 2319x1E	Whole organism	*Thermococcus* sp. 2319x1E grown on cellulose_ replicate 1
ACE_0391_SN06	*Thermococcus* sp. 2319x1E	Whole organism	*Thermococcus* sp. 2319x1E grown on cellulose_ replicate 2
ACE_0391_SN07	*Thermococcus* sp. 2319x1E	Whole organism	*Thermococcus* sp. 2319x1E grown on cellulose_ replicate 3
ACE_0391_SN08	*Thermococcus* sp. 2319x1E	Whole organism	*Thermococcus* sp. 2319x1E grown on cellulose_ replicate 4
ACE_0391_SN09	*Thermococcus* sp. 2319x1E	Whole organism	Thermococcus sp. 2319x1E grown on xylose replicate 1
ACE_0391_SN10	*Thermococcus* sp. 2319x1E	Whole organism	Thermococcus sp. 2319x1E grown on xylose replicate 2
ACE_0391_SN11	*Thermococcus* sp. 2319x1E	Whole organism	Thermococcus sp. 2319x1E grown on xylose replicate 3
ACE_0391_SN12	*Thermococcus* sp. 2319x1E	Whole organism	Thermococcus sp. 2319x1E grown on xylose replicate 4
ACE_0391_SN13_2	*Thermococcus* sp. 2319x1E	Whole organism	Thermococcus sp. 2319x1E grown on xylan replicate 1_2
ACE_0391_SN14_2	*Thermococcus* sp. 2319x1E	Whole organism	*Thermococcus* sp. 2319x1E grown on xylan replicate 2_2
ACE_0391_SN15_2	*Thermococcus* sp. 2319x1E	Whole organism	*Thermococcus* sp. 2319x1E grown on xylan replicate 3_2
ACE_0391_SN16_2	*Thermococcus* sp. 2319x1E	Whole organism	Thermococcus sp. 2319x1E grown on xylan replicate 4_2

LC_Settings

MS device	Thermo Orbitrap Elite
LC device	Thermo Easy-nLC 1000
ion source	Thermo Nanospray Flex
**Analytical column**	Self-packed fused silica capillary with integrated pico frit emitter; New Objectives PF360-75-15-N-5
column diameter	Length (L_C) = 35 cm; ID = 75µm; OD = 360 µm; emitter 15 µm
stationary phase	Reprosil-Pur 120 C18-AQ, Dr. Maisch GmbH
particle diameter (d_p)	1.9 µm
Pore size	120 Å
Column ID	AC62
Column oven	Sonation column oven PRSO-V1
Column oven temp.	45°C
**solvents**	A: 0.1% FA in UPLC water<br>B: 0.1% FA in UPLC ACN
gradient	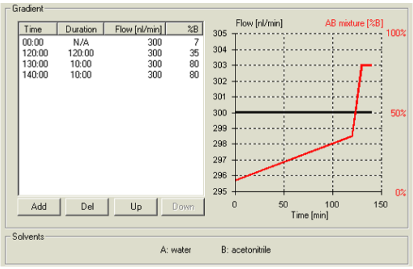

MS_Settings

Project	MS	general	MS1	MS2	MS2	MS3	Comments; special settings
ACE_0391	Elite	Tune v2.7.0.1112 SP2<br>Gradient: 140 min	Analyzer: FT<br>Res.: 60000<br>SR: 300 - 1800<br>AGC: 3×10^6<br>AcT: 50 ms<br>RF: --<br>SF: --<br>DDM: NS	Analyzer: IT<br>Res./ScR: -/rapid<br>SR: Auto<br>AGC: 1×10^4<br>AcT: 50 ms<br>CS: +2 to +7<br>IsM: IT<br>IsW: 2.0<br>Frag.: CID<br>NCE: 35<br>NS: 15			classic orbitrap experiment: MS1 in Orbitrap at high resolution and data dependent MS2 in Iontrap at rapid scan rate. Dynamic exclusion enabled (exclude after n times=1; Exclusion duration (s)= 120; mass tolerance= ± 10ppm)

Note: **FT**= Fourier Transform (Orbitrap); **IT**= Iontrap; **Q**= Quadrupol; **Res.**= max. Resolution at 200 m/z (Lumos) or 400 m/z (Elite) [FWHM (full width at half maximum)]; **ScR**= scan rate for measurements in the IT; **SR**= scan range [m/z]; **AGC**= automatic gain control, max number of acquired ions per measurement; **AcT**= max. Ion acquisition time [ms]; **CS**= charge states used for fragmentation; **IsM**= Isolation mode (Q or IT), MS2 isolation and further is only done in IT; **IsW**= Isolation window [m/z], value followed by scan mode the isolation is based on (MS1, MS2 …) **Frag.**= Fragmentation method; **HCD**= Higher-energy collisional dissociation; **CID**= Collision-induced dissociation; **ETD**= Electron-transfer dissociation**; EThcD=** Electron-Transfer/Higher-Energy Collision Dissociation; **sHCD**= stepped HCD**; NCE**= normalized collision energy; **cycles**: number of MSn recorded or max cycle time; RF= RF Lens [%]; **SF**= Source Fragmentation [V]; **DDM**: Data dependent Mode (cycle time in seconds, CT/[s] or number of scans, NS); **NS**= Number of data dependent scans

ACE_0491

File legend

ACE ID	Organism	Organ/ cell line	Treatment/ experimental setup
ACE_0491_SN01	*Thermococcus* sp. strain 2319x1E		*In vivo* labelling of *Thermococcus* sp. strain 2319x1E with JJB384 grown on xylan_DMSO replicate 1
ACE_0491_SN02	*Thermococcus* sp. strain 2319x1E		*In vivo* labelling of *Thermococcus* sp. strain 2319x1E with JJB384 grown on xylan_DMSO replicate 2
ACE_0491_SN03	*Thermococcus* sp. strain 2319x1E		*In vivo* labelling of *Thermococcus* sp. strain 2319x1E with JJB384 grown on xylan_DMSO replicate 3
ACE_0491_SN04	*Thermococcus* sp. strain 2319x1E		*In vivo* labelling of *Thermococcus* sp. strain 2319x1E with JJB384 grown on xylan_ JJB384 replicate 1
ACE_0491_SN05	*Thermococcus* sp. strain 2319x1E		*In vivo* labelling of *Thermococcus* sp. strain 2319x1E with JJB384 grown on xylan_JJB384 replicate 2
ACE_0491_SN06	*Thermococcus* sp. strain 2319x1E		*In vivo* labelling of *Thermococcus* sp. strain 2319x1E with JJB384 grown on xylan_JJB384 replicate 2
ACE_0491_SN07	*Thermococcus* sp. strain 2319x1E		*In vivo* labelling of *Thermococcus* sp. strain 2319x1E with JJB384 grown on xylose_DMSO replicate 1
ACE_0491_SN08	*Thermococcus* sp. strain 2319x1E		*In vivo* labelling of *Thermococcus* sp. strain 2319x1E with JJB384 grown on xylose_DMSO replicate 2
ACE_0491_SN09	*Thermococcus* sp. strain 2319x1E		*In vivo* labelling of *Thermococcus* sp. strain 2319x1E with JJB384 grown on xylose_DMSO replicate 3
ACE_0491_SN10	*Thermococcus* sp. strain 2319x1E		*In vivo* labelling of *Thermococcus* sp. strain 2319x1E with JJB384 grown on xylose_JJB384 replicate 1
ACE_0491_SN11	*Thermococcus* sp. strain 2319x1E		*In vivo* labelling of *Thermococcus* sp. strain 2319x1E with JJB384 grown on xylose_JJB384 replicate 2
ACE_0491_SN12	*Thermococcus* sp. strain 2319x1E		*In vivo* labelling of *Thermococcus* sp. strain 2319x1E with JJB384 grown on xylose_JJB384 replicate 3

LC_Settings

MS device	Thermo Orbitrap Fusion Lumos
LC device	Thermo Easy-nLC 1200
ion source	Thermo Nanospray Flex
**Analytical column**	Self-packed fused silica capillary with integrated pico frit emitter; New Objectives PF360-75-15-N-5
column diameter	Length (L_C) = 46 cm; ID = 75µm; OD = 360 µm; emitter 15 µm
stationary phase	Reprosil-Pur 120 C18-AQ, Dr. Maisch GmbH
particle diameter (d_p)	1.9 µm
Pore size	120 Å
Column ID	AC80
Column oven	Sonation column oven PRSO-V2
Column oven temp.	50°C
**solvents**	A: 0.1% FA in UPLC water<br>B: 0.1% FA in 80% UPLC ACN and 20% UPLC water
gradient	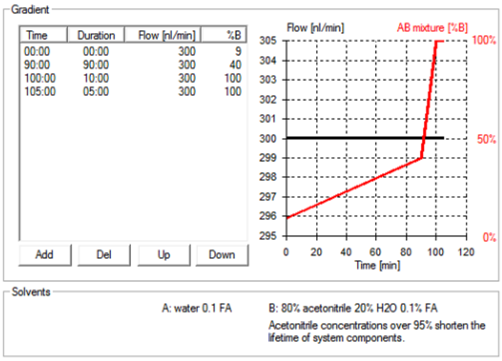

MS_Settings

Project	MS	general	MS1	MS2	MS2	MS3	Comments; special settings
ACE_0491	Lumos	Tune v v3.3.2782.28<br>Gradient: 105 min	Analyzer: FT<br>Res.: 240000<br>SR: 375 - 1500<br>AGC: Standard<br>AcT: Auto<br>RF: 30<br>SF: --<br>DDM: CT/2sec	Analyzer: IT<br>Res./ScR: -/Turbo<br>SR: Auto<br>AGC: Standard<br>AcT: Auto<br>CS: +2 to +7<br>IsM: Q<br>IsW: 1.6<br>Frag.: HCD<br>NCE: 30			classic orbitrap experiment: MS1 in Orbitrap at high resolution and data dependent MS2 in Iontrap at turbo scan rate. Dynamic exclusion enabled (exclude after n times=1; Exclusion duration (s)= 60; mass tolerance= ± 10ppm)

Note: **FT**= Fourier Transform (Orbitrap); **IT**= Iontrap; **Q**= Quadrupol; **Res.**= max. Resolution at 200 m/z (Lumos) or 400 m/z (Elite) [FWHM (full width at half maximum)]; **ScR**= scan rate for measurements in the IT; **SR**= scan range [m/z]; **AGC**= automatic gain control, max number of acquired ions per measurement; **AcT**= max. Ion acquisition time [ms]; **CS**= charge states used for fragmentation; **IsM**= Isolation mode (Q or IT), MS2 isolation and further is only done in IT; **IsW**= Isolation window [m/z], value followed by scan mode the isolation is based on (MS1, MS2 …) **Frag.**= Fragmentation method; **HCD**= Higher-energy collisional dissociation; **CID**= Collision-induced dissociation; **ETD**= Electron-transfer dissociation**; EThcD=** Electron-Transfer/Higher-Energy Collision Dissociation; **sHCD**= stepped HCD**; NCE**= normalized collision energy; **cycles**: number of MSn recorded or max cycle time; RF= RF Lens [%]; **SF**= Source Fragmentation [V]; **DDM**: Data dependent Mode (cycle time in seconds, CT/[s] or number of scans, NS); **NS**= Number of data dependent scans
